# Single cell transcriptome profiling of retinal ganglion cells identifies cellular subtypes

**DOI:** 10.1038/s41467-018-05134-3

**Published:** 2018-07-17

**Authors:** Bruce A. Rheaume, Amyeo Jereen, Mohan Bolisetty, Muhammad S. Sajid, Yue Yang, Kathleen Renna, Lili Sun, Paul Robson, Ephraim F. Trakhtenberg

**Affiliations:** 1 Department of Neuroscience, University of Connecticut School of Medicine, 263 Farmington Ave, Farmington, CT 06030 USA; 2 The Jackson Laboratory for Genomic Medicine, Farmington, CT 06032 USA; 3 Institute for Systems Genomics and Department of Genetics & Genome Sciences, University of Connecticut School of Medicine, Farmington, CT 06032 USA

## Abstract

Retinal ganglion cells (RGCs) convey the major output of information collected from the eye to the brain. Thirty subtypes of RGCs have been identified to date. Here, we analyze 6225 RGCs (average of 5000 genes per cell) from right and left eyes by single-cell RNA-seq and classify them into 40 subtypes using clustering algorithms. We identify additional subtypes and markers, as well as transcription factors predicted to cooperate in specifying RGC subtypes. Zic1, a marker of the right eye-enriched subtype, is validated by immunostaining in situ. Runx1 and Fst, the markers of other subtypes, are validated in purified RGCs by fluorescent in situ hybridization (FISH) and immunostaining. We show the extent of gene expression variability needed for subtype segregation, and we show a hierarchy in diversification from a cell-type population to subtypes. Finally, we present a website for comparing the gene expression of RGC subtypes.

## Abstract (web summary)

Retinal ganglion cells (RGCs) are diverse in cellular function and physiology. This study demonstrates additional RGC heterogeneity using single cell transcriptomic analyses to classify 40 classes of RGCs in early postnatal mice before eye opening.

## Introduction

The complexity of the mammalian central nervous system (CNS) is, in large part, accounted for by an increased number of specialized neuronal types and subtypes, which, in turn, give rise to an even more complex connectome^1^. However, due to the extensive heterogeneity of mammalian neuronal types, many cell types and many more subtypes have not yet been characterized, and many of the fundamental principles of neuronal cell type and subtype biology have yet to be determined^2–5^. Recent advances in droplet-based single-cell RNA sequencing (scRNA-seq) technologies allowed studying the molecular differences between single cells at the cell population level^6,7^, enabling us to address basic questions regarding the biology of neuronal cell types and subtypes. For example: to what extent do cells need to be similar to each other to be a member of a cell type; what extent of variability within a cell type may be sufficient for segregation into subtypes; is there a hierarchy in diversification from a cell type into subtypes; do subtypes from the left and right hemisphere mirror each other; and could stimulus from the environment trigger subtype specification from a neuronal cell type?

We have chosen the retinal ganglion cell (RGC) to address these questions, because more of its subtypes have been identified to date compared to any other major neuronal cell type, and because other broad classes of retinal cell types (e.g., photoreceptors, bipolar, horizontal, amacrine, muller glia) have been studied at a single-cell level. The visual information collected in the retina is pre-processed and passed to the brain by the RGCs, which represent <1% of all retinal cells^8–10^. The RGCs project axons to their targets in the brain, and the left and right eye axons encounter each other in the optic chiasm, where the majority crosses to the contralateral side^11^. Injury to RGCs or their axons could lead to blindness (e.g., glaucoma and various optic neuropathies)^12–14^. Thirty subtypes of RGCs, differing in morphology, localization, function, susceptibility to degeneration, and regenerative capacity, have been identified in the mammalian retina^9,15^ (see Supplementary Discussion). Several subsets of these RGC subtypes have been labeled in transgenic mouse lines, and a number of subtype-specific markers have been described (see Supplementary Discussion). However, the molecular differences between, and the markers unique to, the large majority of RGC subtypes are unknown to date.

A scRNA-seq was recently used to characterize ~44,000 cells from the early postnatal mouse retina^16^. While there are approximately 60,000 RGCs in the mouse retina, they represent <1% of all retinal cell types^8–10^. Not surprisingly, only 432 of the cells profiled in this study were classified as RGCs, which formed a single cluster^16^ and, in retrospect, separated into two categories based on the expression or absence of Opn4 marker^17^ of intrinsically photosensitive RGCs (ipRGCs)^16^. This lack of overt subtype heterogeneity within these scRNA-seq defined RGCs could be because analyzed RGCs were from pre-eye-opening age (postnatal day 12 in mice), after which the visual experience helps shape the maturation of retinal circuitry^18^ and in that process may trigger specification of more subtypes. However, it is also possible that so few RGC subtypes were identified due to a combination of the low number of RGCs captured and the low sensitivity and depth of sequencing of this first generation droplet-based scRNA-seq (e.g., less than half of 432 RGCs in this scRNA-seq data set had over 900 genes detected).

Here, we purified RGCs in large numbers from pre-eye-opening age^3,19–21^, and performed scRNA-seq profiling with an improved, next generation droplet-based method^22^. We detected, on average, 5000 genes at a depth of ~100,000 reads per cell in 6225 RGCs, which represent over 10% of total RGC population. We then used clustering algorithms^22,23^ for classifying the RGCs into subtypes based on their transcriptome profiles. We identified RGC subtypes and markers and predicted the transcription factors (TFs) which may cooperate in specifying RGC subtypes. We also validated RGC subtypes markers Runx1 and Fst and characterized the Zic1 + RGC subtype, which we found enriched in the right eye. We then addressed some of the basic questions in cell type and subtype biology raised above. Finally, we have created a website that provides a platform for analyzing and comparing gene expression profiles in the RGC subtypes.

## Results

### Identification of RGC subtypes

**Fig. 1:**
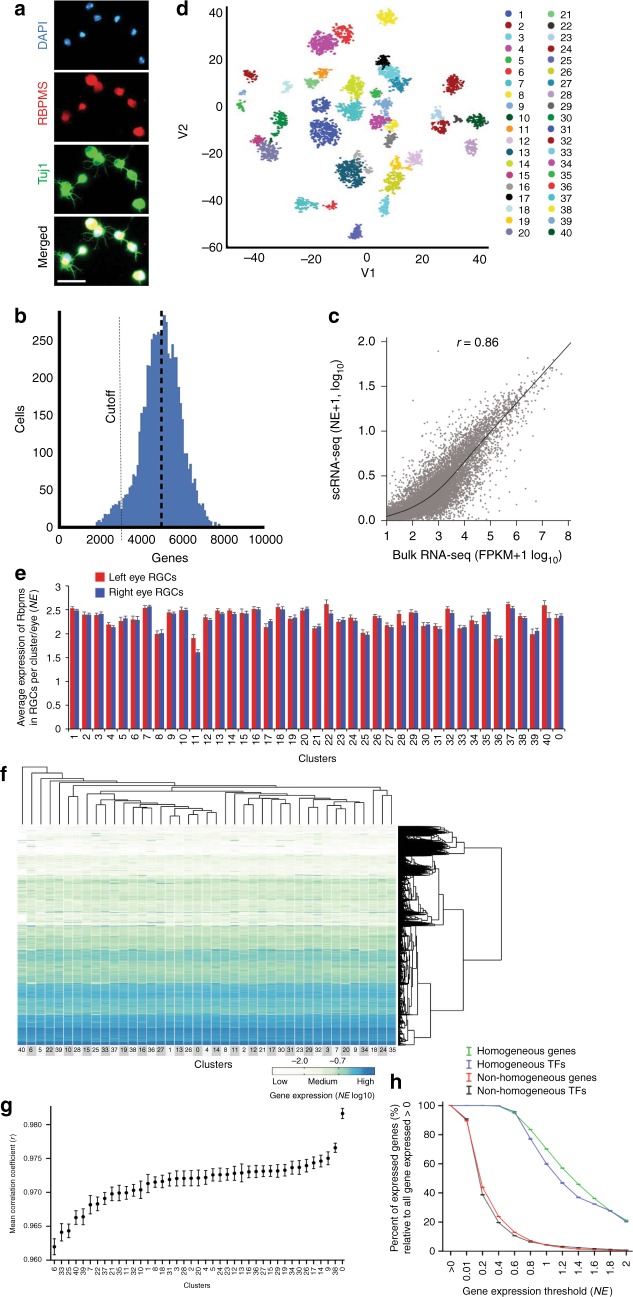
Clustering of single RGCs based on the transcriptomes. **a** Representative image of P5 RGCs immunostained for RGC marker RBPMS and neuronal marker Tuj1, at 12 h in culture after immunopanning (Scale bar, 50 µm). **b** Coverage depth of 5000 genes per cell, on average, was achieved. A sub-peak below 3000 genes per cell was excluded due to poor coverage (a cutoff threshold is indicated by a dashed line). **c** Correlation analysis of gene expression in population of RGCs, as inferred from the scRNA-seq profiling of RGCs (by averaging gene expression from all the cells), and as determined through bulk full-length mRNA-seq of pooled RGCs (Pearson *r* *=* 0.86, 2-tailed, *p* < 0.0001; fit line by LOESS). **d** RGC clusters were identified based on their transcriptome signatures and visualized using CellRanger and CellView pipeline (see Methods section for details). The t-SNE 2D graph, with the 3rd dimension color-coded, shows clusters distribution. **e** RBPMS is highly expressed in all RGC clusters, and in a similar proportion between left and right eyes across all the clusters. **f** Unsupervised *k*-means clustering heatmap and hierarchical clustering dendrogram of 6225 single RGCs gene expression profiles. The vertical distances on each branch of the dendrogram represent the degree of similarity between RGC clusters gene expression profiles. Expression level is color-coded; scale bar is NE log10-transformed following an addition of a pseudocount. **g** Distribution of RGC clusters from less to more similar (left to right) based on mean correlation coefficient between each cluster and every other cluster (mean ± SEM shown, Pearson *r*, 2-tailed). **h** Average percent of genes detected in RGC clusters (*y*-axis) across increasing expression thresholds (*x*-axis) relative to all genes that are expressed > 0 NE either within RGC homogeneous (expressed within 1.5-fold differences between any two clusters) or non-homogeneous (expressed > 1.5-fold difference between at least one cluster and another) genes and TFs categories (mean ± SEM shown; normalized separately within each category for comparison). In all the panels, cluster 0 represents RGCs which did not fit uniquely to any one cluster but expressed RGC markers and were highly correlated with other RGCs overall

We purified RGCs from the left and right eyes of 8 postnatal day 5 (P5) mice by immunopanning^24^ for the RGC surface marker, Thy1^25^. Immunopanning is less stressful to cells than FACS and has allowed for culturing and bulk RNA-seq analysis of purified RGCs^3,19,20^ (Supplementary Figure. 1A-B). Immediately after purification, RGCs from left and right eye were processed separately using the 10× Genomics Chromium platform^22^ (Supplementary Figure. 1C-D). Each cell was sequenced to a depth of ~100,000 reads, resulting in an average of 5000 genes (or 20,000 transcripts, Supplementary Figure. 2E) detected per cell. A sub-peak below 3000 genes per cell was excluded from subsequent analyses due to poor coverage (Fig. 1a, b).

To determine RGC subtypes, we analyzed the RGC transcriptome profiles with t-SNE and DBSCAN cluster determination algorithms, which combine unsupervised hierarchical clustering and dimensionality reduction projections, using the CellRanger and CellView pipeline^22,23^ (Methods section). The analysis of the first clustering round identified a few outlier clusters comprised of 253 cells that highly expressed amacrine or other non-RGC retinal cell markers, which represented a 3.9% contamination with non-RGCs and were excluded from the second round of clustering. Subsequent analyses showed that all RGC subtypes expressed established pan-RGC markers, such as an RBPMS^26–29^ and Tubb3^30^ (Fig. 1e and Supplementary Figure. 2C; also see below). In total, 6225 cells that expressed RGC markers (with over 2500 from left and right eye each) were used in the final clustering round and subsequent downstream analyses. Thy1, the gene encoding the epitope we used for immunopanning, was highly expressed in all RGCs but more in some clusters than in others. The variance in Thy1 level of expression was not stochastic, as separately processed left and right RGCs expressed it in similar proportions in all the clusters (Supplementary Figure. 2A). However, there was no association between the level of Thy1 expression and the number of RGCs comprising clusters (Supplementary Figure. 2B), suggesting that the varying level of Thy1 expression between the subtypes did not appear to bias RGC selection (as also implied from prior studies^24,31^). The data quality is also demonstrated by strong correlation (*r* = 0.86, Pearson) between polyA-selected RNA-seq of bulk pooled RGCs of the same age^3^ and the average gene expression of single RGCs (Fig. 1c). Thus, a combination of depth of sequencing and the large numbers of RGCs provided a high-quality resource for comprehensive classification of the RGCs into subtypes.

We found that the RGC population segregated into 40 clusters (Fig. 1d; lists of genes enriched in each cluster are in Supplementary Data 1). Because we have predicted more than 30 known RGC subtypes from pre-eye-opening RGCs, our data suggests that initial RGC subtype specification does not require visual stimulation^18,32^ and that changes in gene expression during further maturation^33^ may be fine-tuning or further subdividing already specified subtypes, as well as affecting RGC housekeeping functions, such as inactivation of intrinsic axon growth capacity^34–36^. Remarkably, recent classification of adult RGCs by electrophysiological properties also identified 40 categories^37^, raising the hypothesis that the molecular differences we found between 40 RGC subtypes may underlie these distinct electrophysiological functions.

### Characteristics of cell type and subtype

We next investigated the extent of similarity between the cells that a membership in RGC cell type would entail, and the extent of variability within a cell type that could be sufficient for segregation into subtypes. A heatmap of the hierarchical cluster analysis showed that RGC subtypes, overall, have a similar gene expression profile (Fig. 1f), which is confirmed by the high correlation (*r* > 0.9, Pearson) between any two clusters (Supplementary Figure. 2D). However, within the remaining variability, some clusters are more similar to each other than others (Fig. 1g), reflecting heterogeneity of RGC subtypes captured by the cluster determination algorithms. These results demonstrate that, as a cell type, RGCs are very similar to each other, although quite different from other cell types, as we have recently reported^3^. Nevertheless, the narrow window of variability is sufficient to distinguish between 40 RGC subtypes at a molecular level.

To determine the genes and TFs that define an RGC as a cell type, we analyzed gene expression patterns that are either expressed at similar levels (within 1.5-fold) in all or only some RGC subtypes. When including genes with low expression values (i.e., anything > 0 normalized expression (NE) in the data set), we found much fewer TFs (53) and other genes (1699) that define an RGC as a cell type, compared to many more TFs (655) and other genes (11,193) whose expression differs between the RGC subtypes (Fig. 1h, showing only percentages normalized within each category, see figure legend; TF IDs and NE are in Supplementary Data 2). However, this difference was lost at >0.8 NE threshold, with more TFs (41) and other genes (1419) defining an RGC as a cell type, compared to TFs (41) and other genes (807) whose expression differs between the RGC subtypes (these differences in numbers are not seen in Fig. 1h, because it shows only percentages per category). These data suggest that, while more TFs and genes are involved in differentiating between RGC subtypes than in maintaining an RGC as a cell type, these genes that differentiate between RGC subtypes are expressed highly only in some subtypes and consequently present as low expressed when averaged across all RGCs at the cell-type level. Furthermore, as the genes that differentiate between RGC subtypes are expressed predominantly only at low levels in most of the subtypes, fold-change differences between them (relative to the total expression range in the data set) are not weighing as much to decrease the correlation coefficients reported above.

### Laterality of RGC subtypes

**Fig. 2:**
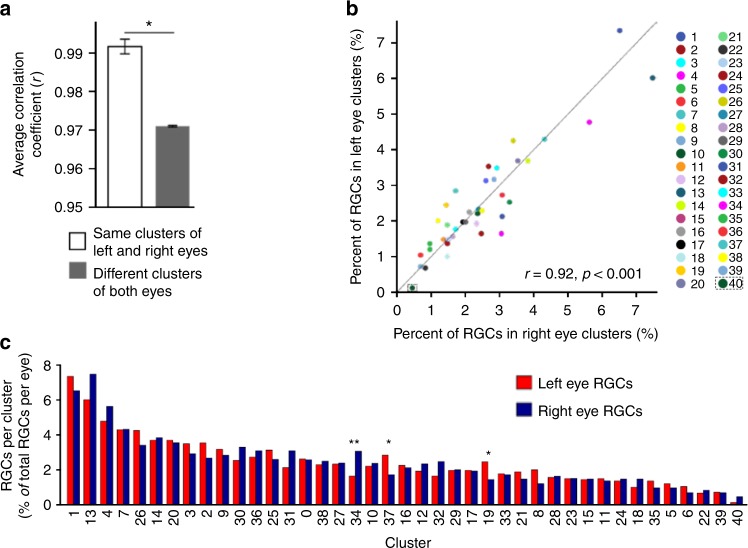
Distribution of RGC subtypes by left/right eye. **a** Mean correlation coefficient between gene expression profiles of the same clusters in the left and right eyes is significantly higher than mean correlation coefficient between gene expression profiles of different clusters from both eyes (mean ± SEM shown, Pearson *r*, 2-tailed; *p < 0.0001 by independent samples *t*-test, 2-tailed). **b** Percent of RGCs per cluster is shown relative to the total RGCs in the left (red) or right (blue) eye. The bars are ranked from higher to lower based on the average percent of cells per cluster from both eyes. Significant (*p < 0.01 and **p < 0.001) clusters enrichments per eye are shown (statistical analysis described in Methods section). **c** Percent of RGCs comprising left and right eye clusters is highly correlated, *r* = 0.92, *p* < 0.001 (Pearson, 2-tailed)

We then asked whether RGC clusters in the left and right eye mirror each other, and also investigated why cluster 40 diverged furthest from all the other clusters in the dendrogram (Fig. 1f). We found that the mean correlation coefficient between gene expression profiles of the same clusters from the left and right eyes (*r* = 0.99, Pearson) was significantly higher than that of different clusters from both eyes (*r* = 0.97, Pearson) (Fig. 2a). There were exceptions, however, as subtypes 40 and 34 were enriched (3.8-fold and 1.86-fold, respectively) in the right eye, and, to a lesser extent, subtypes 19 and 37 were enriched (1.7-fold and 1.6-fold) in the left eye (Supplementary Table 1 and Fig. 2b). Because by most subtypes were distributed proportionally, with percent of RGCs comprising left and right eye subtypes highly correlated (*r* = 0.92, *p* < 0.001; Pearson, 2-tailed; Fig. 2b, c and Supplementary Table 1), subtype 40 being the most disproportionally represented in one eye also diverging the furthest in the dendrogram (Fig. 1f), suggests that there may be an association between the asymmetric representation of this cluster and divergence based on gene expression. These data show that, overall left and right eye RGC subtype transcriptomes mirror each other and are distributed proportionally between the eyes. These data also demonstrate that a few RGC subtypes are overrepresented in one eye, suggesting that some eye functions may be predominant in one eye.

### RGC subtype distribution

**Fig. 3:**
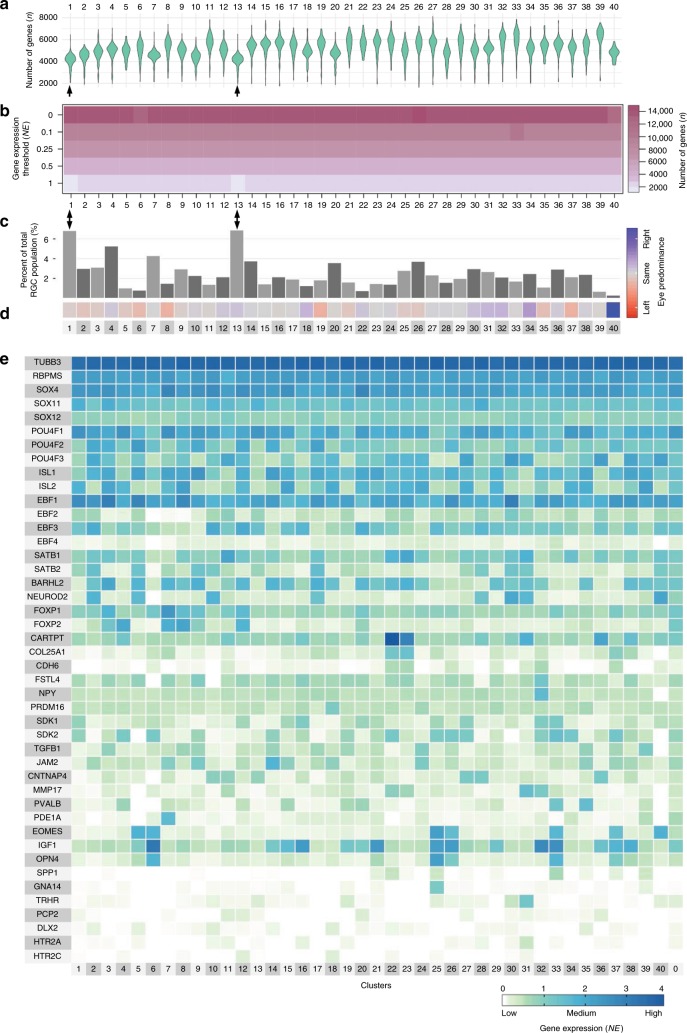
Global properties of subtype transcriptome and the known RGC markers. **a** Violin graph showing average number and distribution of genes expressed per cluster as probability density. **b** Proportion of genes number expressed across different ranges, from low to highly expressed genes. **c**, **d** Percent of RGCs per cluster (**c**) and color-coded predominance of the cluster in left (red) or right (blue) eye (**d**). Subtypes 1 and 13 indicated by arrowheads in (**a**–**c**) are the lighter intensity boxes in (**b**) at the 1 NE threshold. **e** Heatmap of the known RGC markers shows that Tubb3, RBPMS, and Sox4 have the most homogenous expression across all RGC subtypes. Most of the known RGC markers show strong expression in more than one cluster

Next, we asked whether there is a predominant subtype that may be central to the RGC as a cell type. We found that the proportion of RGCs comprising a subtype relative to the total population of RGCs ranged from 6.93% (subtype 1) to 0.71% (subtype 39), with the exception of subtype 40, which represented 0.29% but was largely accounted for by the right eye RGCs (Supplementary Table 1 and Fig. 2b). Approximately 2.4% of RGCs, in similar proportion from each eye, did not associate significantly with any specific subtype by the clustering algorithm and were grouped under cluster 0 (Fig. 1e–g). Cluster 0 RGCs were highly correlated (*r* > 0.9, Pearson; Fig. 1g) with all other clusters and expressed the RGC marker, RBPMS^26–29^, at a level comparable to other RGC subtypes (~2.3 NE) (Figs. 1e and 3e). This suggests that these might be atypical RGCs that are most similar to subtype 26 (based on the cluster analysis dendrogram; Fig. 1f), or that these RGCs are less mature and may form additional subtypes later in development. These data show that, while the subtypes vary in the proportion of RGCs that comprise them, there was no substantially overrepresented subtype, suggesting that the RGC’s role as a cell type overall may not have a key driving subtype but rather similarly depend on multiple subtypes.

### Global properties of subtype transcriptome

Because cell types differ in global properties of the transcriptome^3^, we asked whether subtypes also differ in global properties of the transcriptome. We analyzed the average number and distribution of genes expressed per cluster as a probability density^38^ and found that subtypes 1 and 13 had the highest number of cells expressing the fewest genes compared to the other subtypes (followed by subtypes 7 and 10), whereas subtypes 33 and 39 had the highest number of cells expressing the most genes compared to the other subtypes (followed by subtypes 11 and 32) (Fig. 3a). We then analyzed whether RGC subtypes differ in the proportion of highly expressed genes and found that, except subtypes 1 and 13, all the subtypes had a very similar proportion of highly expressed genes (Fig. 3b). Subtypes 1 and 13 were also comprised of more RGCs than other subtypes, 6.93% and 6.75%, respectively (Fig. 3c), and belong to the same intermediate subpopulation 4 (see below) These subtypes were distributed similarly in the left and right eyes (Fig. 3d). Only minor differences in the proportion of highly expressed genes (Fig. 3b) and only in 2 subtypes (1 and 13) is in contrast to significant differences between cell types (e.g., RGC, cortical neuron, oligodendrocyte, etc.) in proportion of highly expressed genes^3^. Because the global size of the transcriptome is related to cell size^3,39^, we explain in the Supplementary Discussion how these data raises the hypothesis that subtypes 1 and 13 could be midget RGCs, and subtypes 33 and 39 could be αRGCs.

### RGC subtype-specific markers

To further investigate the RGC subtypes, we asked to what extent they could be explained by the known RGC markers, including pan-markers that label all or many RGC subtypes (RBPMS, Tubb3, Pou4f1-3, Sox4, Sox11-12, Isl1-2; see Supplementary Discussion), as well as those reported to be subtype or subset-specific (Foxp1-2, Cartpt, Col25a1, Fstl4, NPY, Sdk1-2, Mmp17, Jam2, Pvalb, Opn4, Cdh6, NeuroD2, Htr2a, Htr2c, Igf1, Pde1a, Gna14, Trhr, Pcp2, Barhl2, Eomes/Tbr2, Dlx2, Ebf1-4, Satb1-2, Cntnap4, Prdm16; see Supplementary Discussion). We found that most of the known markers were enriched in more than one cluster but were restricted to various RGC subpopulations. Only Jam2, NPY, Pde1a, Trhr, and Gna14 were enriched in single clusters (Fig. 3e). This data raises the hypothesis that there might be a hierarchy in RGC subtype segregation, from subpopulations to subtypes. Moreover, two of the most prevalent subtypes, 1 and 13, were not enriched with any of the known subtype-specific markers (Fig. 3c, e), highlighting the need for more specific RGC subtype markers.

**Fig. 4:**
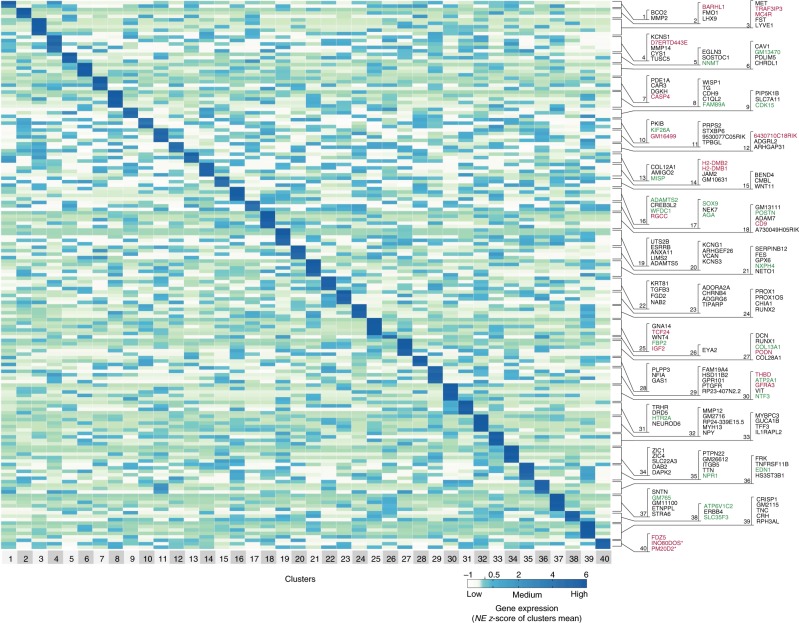
Heatmap signatures of genes enriched in RGC subtypes. Enriched genes selection criteria: expression > 1.8-fold relative to every other cluster, at *p*-value ≤ 0.05 (except for two cluster 40 enriched genes which did not pass the *p*-value threshold, indicated by *, as detailed in the Methods section), and minimal expression > 0.05 NE. Genes with *p* ≤ 0.05 by *t*-test and Mann–Whitney *U* independent samples tests are shown in black font. Genes with *p* ≤ 0.05 by *t*-test and *p* ≤ 0.1 by Mann–Whitney *U* test are shown in green font. Genes with *p* ≤ 0.05 by either test but *p* > 0.1 by the other are shown in red font. Color-coded scale bar of gene expression indicates *z*-scores after normalizing to all the clusters (Methods section). Other genes enriched per cluster only based on the first two criteria are shown in Supplementary Data. 1

**Fig. 5:**
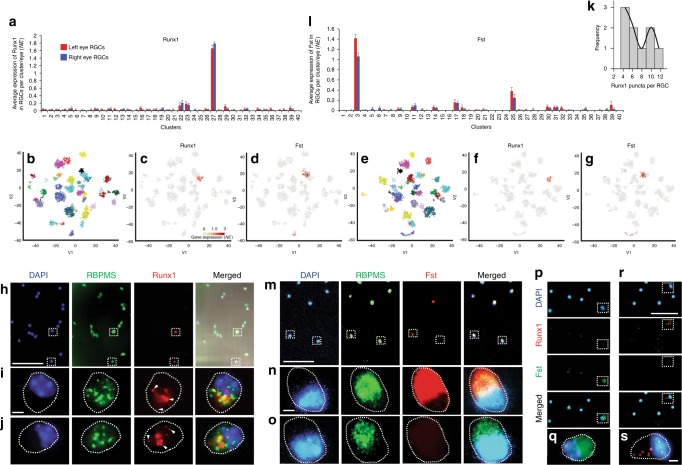
Runx1 and Fst label RGC subtypes. **a** Runx1 expression is enriched in the RGC subtype 27 (mean ± SEM shown). **b**–**g** The t-SNE graph with V1,V2 2D view and color-code as in Fig. 1d, shows clusters distribution in (**b**) and just the expression of Runx1 in (**c**) and Fst in (**d**). An alternative V1,V3 2D view t-SNE graph shows clusters distribution in (**e**) and just the expression of Runx1 in (**f**) and Fst in (**g**), demonstrating clearer separation between the clusters 27 and 3, as well as their markers (Runx1 and Fst), in the 3rd dimension (which is color-coded in the V1,V2 2D view). **h** Representative image of purified P5 RGCs at 12 h in culture probed by FISH for Runx1, an RGC marker RBPMS, and DAPI, as marked. **i**, **j** Insets of two Runx1+ RGCs outlined with dashed box in the upper panels (in the middle **i** and on the bottom **j**) are shown enlarged below (cell soma outlined with dashed line). Merged image in the upper panel also includes brightfield, which was used to outline cell soma shape in the insets. Granularized signal (arrowheads) in the insets is a property of the single-molecule sensitivity FISH kit. **k** Interpolation line shows a higher distribution peak over RGCs with fewer Runx1 puncta compared to RGCs with more puncta. **l** Fst expression is highly enriched in the RGC subtype 3 (mean ± SEM shown). **m** Representative image of purified P5 RGCs at 12 h in culture immunostained for Fst, an RGC marker RBPMS, and DAPI, as marked. **n**, **o** Insets of Fst+ and Fst− RGCs outlined with dashed box in the upper panels (in the middle **n** and on the bottom **o**) are shown enlarged below (cell soma outlined with dashed line). **p**–**s** Representative images of purified P5 RGCs at 12 h in culture probed by FISH for Runx1, and then immunostained for Fst and DAPI, as marked. Insets of Runx1−/Fst+ (**p**) and Runx1+/Fst− (**r**) RGCs outlined with dashed box in the upper panels are shown enlarged below (**q** and **s**, respectively; cell soma outlined with dashed line). (Scale bars: upper panels, 100 µm; lower panels, 5 µm)

To identify subtype-specific markers, we performed a differential expression analysis and predicted markers uniquely enriched in each subtype (Fig. 4). Five of the markers were previously reported as subtype-specific: Jam2 (subtype 14), NPY (subtype 32), Pde1a (subtype 7), Trhr (subtype 31), and Gna14 (subtype 25); see Supplementary Discussion. Twenty of the markers were shown to be expressed in subsets of RGCs in previous screens^29,40,41^ (Supplementary Table 2); another 108 genes, also shown to be expressed in subsets of RGCs in those screens, were enriched in more than one subtype each (Supplementary Figure. 3). We then used fluorescent in situ hybridization (FISH) in purified RGCs to validate the subtype 27-specific marker, Runx1 (Figs. 3e and  5a–g), which was previously shown by immunostaining to label a subset of RGCs^41^ (Supplementary Figure. 3). We detected the Runx1 signal only in a small fraction of RGCs (RBPMS+ cells; Fig. 5h-j). Cluster 27, which highly expresses Runx1 (median 1.8 NE), represents 2.36% of all RGCs; however, 4.85% of RGCs from other clusters also express Runx1 but at a low level (median 0.6 NE). Thus, the total of enriched (cluster 27) and low-expressing (other clusters) Runx1 RGCs is 7.21%. We quantified all Runx1+/RBPMS+ RGCs as percent of total RGCs (RBPMS+), even those with low Runx1 signal (i.e., fewer Runx1 puncta detected per RGC, which comprise marginally higher proportion compared to RGCs with more Runx1 puncta; Fig. 5k). We found that their mean of 8.4% (±3 SD) was not significantly different (*p* = 0.56 by One-Sample *t*-test; *N* = 3, with >25 cells counted per experiment) from the predicted total (population mean) of 7.21%. We also validated the predicted RGC subtype 3-specific marker, Fst (Fig. 5b–g, l). Cluster 3, which highly expresses Fst (median 1.3 NE), represents 3.21% of all RGCs; however, 2.96% of RGCs from other clusters also express Fst at a low level (median 0.7 NE). We quantified Fst+/RBPMS+ RGCs as percent of total RGCs (RBPMS+ cells; Fig. 5m–o). We did not count cells with low Fst signal intensity because it was not possible, with immunostaining, to reliably distinguish a low signal from noise. We found that their mean of 2.8% (±0.3 SD) was not significantly different (*p* = 0.13 by One-Sample *t*-test; *N* = 3, with >50 cells counted per experiment) from the predicted cluster 3 population mean of 3.21%. To confirm that Runx1 and Fst do not label the same RGCs, we probed RGCs by FISH for Runx1, and then immunostained for Fst. These are low abundance subtypes and we did not encounter both subtypes in the same field of view, but the representative images of Runx1−/Fst+ (Fig. 5p, q) and Runx1+/Fst− (Fig. 5r, s) RGCs are from the same well (purity of RGC culture was confirmed in another well as above). These data suggest that Runx1 and Fst are subtype 27 and subtype 3-specific markers, and lends support to the validity of the other subtype-specific markers we predicted.

### Zic1 is a marker of the right eye-enriched RGC subtype 34

**Fig. 6:**
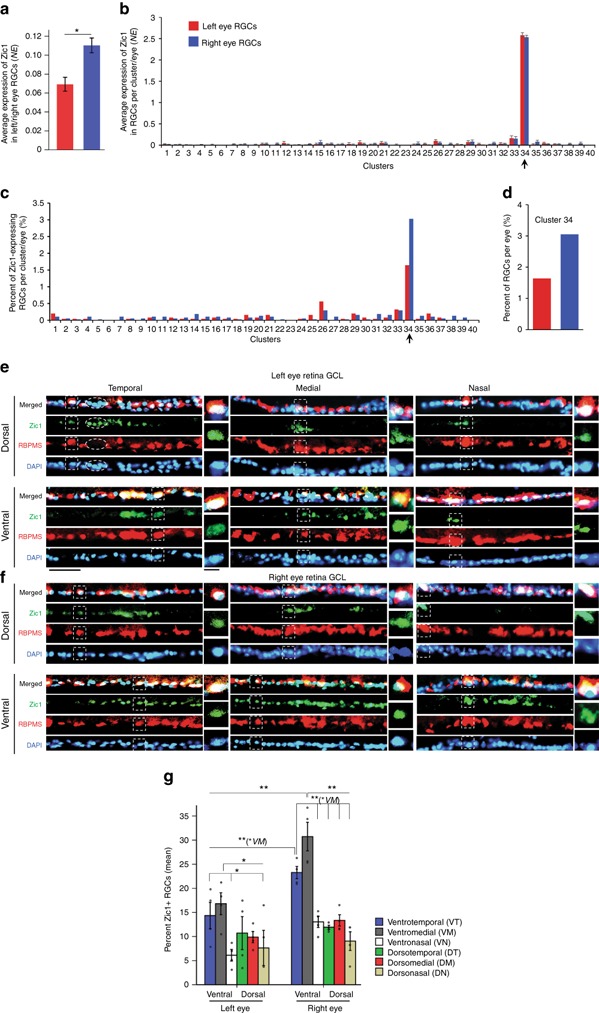
Validation of Zic1 as a marker for the right eye-enriched RGC subtype 34. **a** Average expression of Zic1 is higher in the right eye RGCs compared to the left eye RGCs (mean ± SEM shown; independent samples *t*-test, *p < 0.0001). **b** Average expression of Zic1 per RGC is similar between right and left eye RGCs in cluster 34, which is enriched for Zic1 (mean ± SEM shown; arrow indicates subtype 34). **c**, **d** Percent of RGCs per cluster that express Zic1 is shown relative to the total RGCs in the left (red) or right (blue) eye. Arrow indicates subtype 34 in which Zic1 is expressed in almost twice as many right eye RGCs compared to the left eye (**c**), consistent with the percent of right eye RGCs in subtype 34 being almost twice higher compared to left eye RGCs, relative to the total RGCs in each eye (**d**). **e**, **f** Representative images of retinal cross-sections ganglion cell layer (GCL) from the left (**e**) and right (**f**) eyes of mature mice, immunostained as marked for an RGC marker RBPMS, nuclear marker DAPI, and Zic1. Enlarged examples of Zic1+/RBPMS+/DAPI+ RGCs from regions outlined in dashed line box are shown in insets on the sides, demonstrate that Zic1 signal colocalizes with DAPI in the nucleus, as expected for a TF. An example of Zic1+/RBPMS−/DAPI+ (possibly displaced amacrine) cells in the GCL is outlined in dashed line oval (**e**). Scale bars as marked. **g** Quantifications of Zic1+/RBPMS+ RGCs as percent of total RGCs in different retinal regions show significant enrichment of Zic1+ RGCs in the right eye overall and in the ventrotemporomedial retinal regions in both eyes (mean ± SEM shown; *p < 0.05, **p *<* 0.01 by ANOVA with post hoc LSD pairwise comparisons; *N* *=* 4 for left and right eye each, with different retinal regions quantified from each eye, shown as dots plot overlaying the bars, as detailed in the Methods)

Next, we validated Zic1 as a marker of subtype 34 (Fig. 6), which is 1.86-fold enriched in the right eye (Fig. 2b; although cluster 40 had higher fold enrichment in one eye, it had fewer cells than any other cluster, and, as a result, its enrichment was not statistically significant). We found that Zic1 expression was significantly higher (1.6-fold) in the right eye RGCs, overall (Fig. 6a); however, Zic1 expression per RGC was similar between the left and right eye RGCs comprising subtype 34 (Fig. 6b). Consistent with the higher percent of right eye RGCs in subtype 34 (Figs. 2b and 6d), the percent of RGCs that express Zic1 in subtype 34 was also almost twice as high in the right eye (Fig. 6c). To test whether Zic1+ subtype 34 RGCs are enriched in the right eye through maturation and to characterize spatial distribution of Zic1+ RGCs in the left and right eye retinas, we analyzed mature retinal sections from the right and left eyes by immunostaining for Zic1 and RBPMS (RGC marker). We found that the percent of Zic1+/RBPMS+ RGCs was significantly higher in the right eye, although Zic1 was also expressed in non-RGC (RBPMS−) cells (Fig. 6e–g). We then quantified the ratio of right to left eye Zic1+/RBPMS+ RGCs as percent of total RGCs (RBPMS+ cells; Fig. 6e, f). We found that the mean ratio of 1.61 (±0.36 SD) was not significantly different (*p* = 0.25 by One-Sample *t*-test; *N* = 4 sets of left and right eyes) from the predicted cluster 34 population enrichment ratio of 1.86 (right to left eye). Although histological analysis showing that only a subset of RGCs (RBPMS+ cells) are Zic1+ supports the prediction that Zic1 labels an RGC subtype, the percent of histologically quantified Zic1+ RGCs (Fig. 6g) was higher than scRNA-seq-predicted percent of all Zic1-expressing RGCs (Fig. 6c); and whereas RGCs from cluster 34 express Zic1 highly (median 2.62 NE) and those from other clusters express Zic1 at a lower level (median 0.68 NE), we were not able to reliably discern the different levels of Zic1 expression by immunostaining. Discrepancies between RNA-seq predictions of transcript expression and protein levels are common^42,43^, and may explain the differences we observed. However, as there were no discrepancies between scRNA-seq and immunostaining in the ratio of Zic1 expression in the right to left eye RGCs, we also quantified spatial distribution of Zic1+ RGCs in the retinas. We found that Zic1+ RGCs were significantly enriched in the ventrotemporamedial retina in both eyes (Fig. 6e–g). Ipsilaterally projecting RGCs are located in the ventrotemporal region of the mouse retina^44,45^, and recent microarray analysis of contralateral and ipsilateral projecting RGCs showed that Zic1 expression was enriched in the ipsilaterally projecting RGCs^46^, thus suggesting that subtype 34 may include ipsilaterally projecting RGCs.

### RGC subtype diversification

**Fig. 7:**
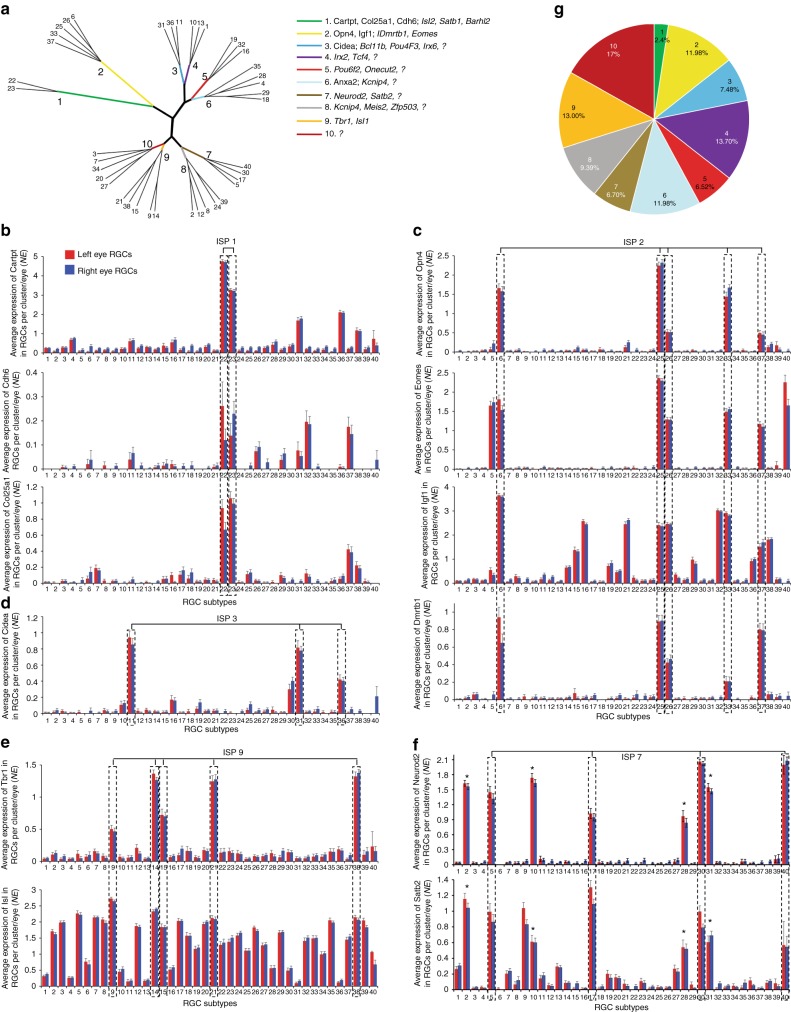
RGC subtypes diversification. **a** Circular phylogenetic tree-style diagram of RGC subtypes hierarchical diversification. The distance of a branch from the center point represents the extent of its divergence, and ramification of the branches represents the hierarchical relationship between RGC subtypes transcriptome profiles. Genes enriched in color-coded intermediate branches, that represent the ISPs, are shown on the side. TFs are italicized, and where a combination of TFs is not sufficient to explain all the subtypes comprising the ISP, a “*?*” represents yet undetermined TF or epigenetic/TF regulator that may participate in specifying these ISPs. **b**–**f** Expression profiles of genes, including TFs, which are enriched in specific ISPs are shown, as marked. **g** The pie graph shows prevalence of the ISPs relative to the total RGC population

Because we found that some RGC markers are enriched in several subtypes whereas others are subtype-specific, we investigated the hierarchy of RGC subtype diversification, from population to subpopulations and then to subtypes. We identified four superclusters, comprised of ten intermediate clusters (the main and the intermediate branches of the phylogenetic-style tree) that represent RGC subpopulations, which are further diversified into 40 subtypes (Fig. 7a). Some of the established RGC markers (Fig. 3e) were enriched in the intermediate clusters, suggesting that they label defined intermediate subpopulations (ISP) of RGCs (Fig. 7a). For example, Cartpt, Cdh6, and Col25a1, the markers of ON-OFF direction-selective RGCs (ooDSGCs)^47^, were enriched in ISP 1, although also expressed at lower levels in some clusters from other ISPs (Fig. 7b). Opn4, Eomes, and Igf1, the markers of melanopsin-expressing ipRGCs^16,17,48^, were enriched in, and Dmrtb1 we identified was unique to, ISP 2 (Fig. 7c; see details below). Cidea, detected in a subset of RGCs in a previous screen^40^ (Supplementary Figure. 3), was enriched in ISP 3, and a marker, Tbr1, was enriched in ISP 9 (Fig. 7d, e). Just as there were no drastically overrepresented clusters based on the proportion of RGCs that comprise them (as discussed above), there were also no substantially overrepresented ISPs; however, ISP 1 was underrepresented, accounting for only 2.4% of total RGCs (Fig. 7g). The hierarchical scheme of RGC subtype diversification raises the hypothesis that, during development, RGCs may first differentiate into 4 major subpopulations, then into 10 ISPs, and finally into 40 subtypes.

### RGC subtype-specific TF combinations

We then asked whether the hierarchical scheme of RGC subtype diversification could be explained by combinations of enriched TFs, which may cooperate in regulating different stages of RGC differentiation. We identified TFs co-enriched in the ISPs (i.e., in all clusters comprising an ISP), as well as performed combinatorial analysis (Methods section) of the TFs that are enriched as unique combinations in the ISPs (i.e., in all clusters comprising an ISP) (Fig. 7a). Several of these TFs are well-known to label subsets of RGCs, and some were shown in previous screens to be expressed in subsets of RGCs^29,40,41^ (Fig. 3e and Supplementary Table 2). For example, Eomes^17^, the marker of ISP 2 ipRGCs (see more below), is also expressed in subtypes 5 and 40, which belong to other ISPs. However, Dmrtb1 is expressed exclusively in ISP 2 subtypes and may cooperate with Eomes in predefining the transcriptional landscape of ISP 2 for further specification into subtypes by subtype-specific TFs (Fig. 7c). In another example, Tbr1 is enriched uniquely in ISP 9, which is also enriched for Isl1 but not uniquely; however, these TFs may cooperate with each other in predefining ISP 9 for further specification into subtypes by subtype-specific TFs (Fig. 7e). For some ISPs we found co-enriched non-unique combinations of TFs, such as Neurod2 and Satb2 in the ISP 7 (Fig. 7f). Neurod2 and Satb2 are known to be involved in specification of RGC subtypes (Fig. 4)^41,49^; however, they are also co-enriched in subtypes 2, 10, 28, and 31 (marked by “*” in Fig. 7f) from other ISPs, and thus may cooperate with yet another TF or epigenetic/TF regulator (marked by “*?*“, Fig. 7a) to specify the ISP 7.

**Fig. 8:**
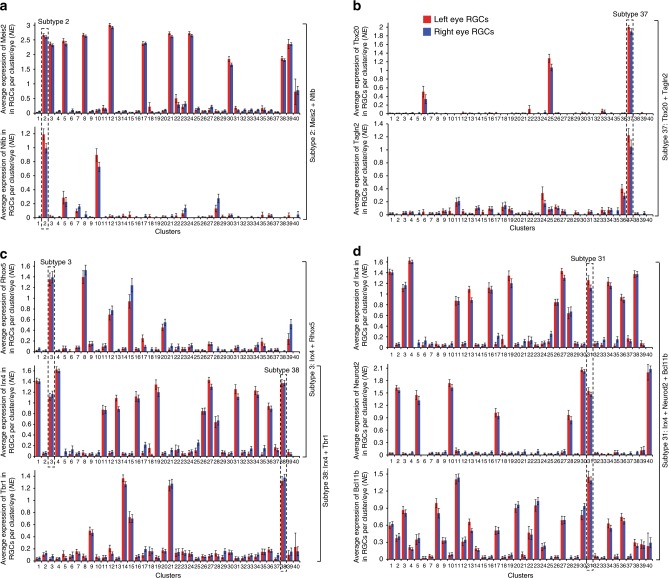
Examples of RGC subtype-specific TF combinations. **a**, **b** 2-way combinations of TFs (**a**), and a TF and transcriptional regulator Tagln2 (**b**), uniquely enriched in RGC subtypes. **c** Different 2-way TF combinations involving the same TF uniquely enriched in different RGC subtypes. **d** Three-way TF combination uniquely enriched in an RGC subtype

Next, we identified TF combinations that are uniquely enriched in RGC subtypes as a combination but not individually (Table 1; see Methods for the computational pipeline). A number of these TFs are well-known to label subsets of RGCs, and several were shown in previous screens to be expressed in subsets of RGCs^29,40,41^ (Fig. 3e and Supplementary Table 2). Subtype 34 had uniquely enriched single TF (Zic1) (Fig. 7b), and subtype 27 had two TFs enriched individually (Runx1 and Mef2c). Most of the other clusters were uniquely enriched by 2-way TF combinations (Fig. 8a–c), and some were uniquely enriched by 3-way TF combinations (Fig. 8d). Some of the clusters could not be completely explained even by up to 5-way combinations, due to an overlap with other subtypes. We report combinations of TFs, including TF regulators (marked by “*”), for all 40 subtypes. Those that are not completely unique (marked by “*?*“) may cooperate with yet another TF or epigenetic/TF regulator to specify these subtypes (Table 1). Taken together, these data suggest that, the hierarchical scheme of RGC subtypes diversification could be explained by unique combinations of TFs or their regulators, some of which are yet to be identified.

**Table 1:** TF combinations uniquely enriched in RGC subtypes

**Subtype**	**TF1**	**TF2**	**TF3**	**Compared to other clusters**	**Between other clusters**
1	Irx3	Tox	?	0.00	0.43
2	Meis2	Nfib		0.00	0.38
3	Rhox5	Irx4		0.00	0.35
4	Foxp2	Gfi1		0.00	0.62
5	Eomes	Meis2	Pou3f1	0.00	0.31
6	Eomes	Nr2f1		0.02	0.61
7	Arid5b	Foxp2		0.00	0.46
8	Rhox5	Foxp2	?	0.00	0.59
9	Foxp1	Satb2	?	0.00	0.39
10	Nfib	Ldb2*		0.00	0.40
11	Satb1	Bcl11b	?	0.05	0.26
12	Foxp1	Neurod1		0.03	0.39
13	Irx4	Ldb2*	?	0.03	0.31
14	Irx6	Tbr1	?	0.00	0.24
15	Tbr1	Isl2		0.00	0.28
16	Irx3	Nr2f2		0.00	0.48
17	Zbtb20	Meis2		0.05	0.35
18	Mafb	Prdm16		0.05	0.63
19	Etv1	Irx3	?	0.02	0.46
20	Bcl11b	Ebf3	?	0.00	0.23
21	Tbr1	Meis2	Sox6	0.00	0.34
22	Satb1	Ctbp2*		0.05	0.38
23	Satb1	Tshz3	?	0.00	0.35
24	Mafb	Tshz3		0.01	0.47
25	Tbx20	Nrg1		0.00	0.62
26	Irx1	Pou6f2	?	0.00	0.19
27	Runx1	Mef2c		0.00	0.70
28	Mafb	Nfia		0.00	0.55
29	Zfhx3	Isl2	?	0.10ª	0.16
30	Neurod2	Crabp1*		0.00	0.38
31	Neurod2	Irx4	Bcl11b	0.00	0.31
32	Nr2f2	Hipk2		0.00	0.59
33	Esrrg	Irx1		0.00	0.61
34	Zic1^b^				
35	Kcnip4	Nfic		0.00	0.25
36	Irx3	Zbtb16	?	0.03	0.38
37	Tbx20	Tagln2*		0.00	0.72
38	Irx4	Tbr1		0.00	0.36
39	Mafb	Kcnip2		0.00	0.53
40	Pou4f3	Eomes	Bhlhe22	0.00	0.15

Different combinations between 46 TFs and 4 transcriptional regulators are sufficient to explain the differences between RGC subtypes. Where the combination explains RGC subtype differentiation from most but not all the other subtypes, a “*?*” is shown in place of yet to be identified TF/TF regulator. Transcriptional regulators in the combinations with TFs are marked by “*”. Statistical interaction of the predicted TFs enrichment as a combination in respective clusters relative to every other cluster was analyzed by Repeated Measures ANOVA with Tukey’s HSD post hoc for pairwise comparisons (SPSS), and was significant at *p* ≤ 0.05 for all RGC subtypes (average *p*-values are shown) with sphericity assumed, except subtype 29, for which the combination does not explain differentiation from all but only from most other subtypes and therefore was significant only at *p* = 0.1 (marked by “a”). Subtype 34 (marked by “b”) is explained by a single TF (for which statistically significant *p*-value was calculated using a different approach, see Fig. 4), and therefore a *p*-value for combination is not applicable for this subtype. For comparison, TFs’ statistical interaction in all other clusters compared to each other (excluding the cluster in which the combination was predicted to be enriched) was analyzed similarly, and it was not significant for any subtype (average *p*-values are shown).

### RGC subtypes enriched for axon regeneration-regulating genes

**Fig. 9:**
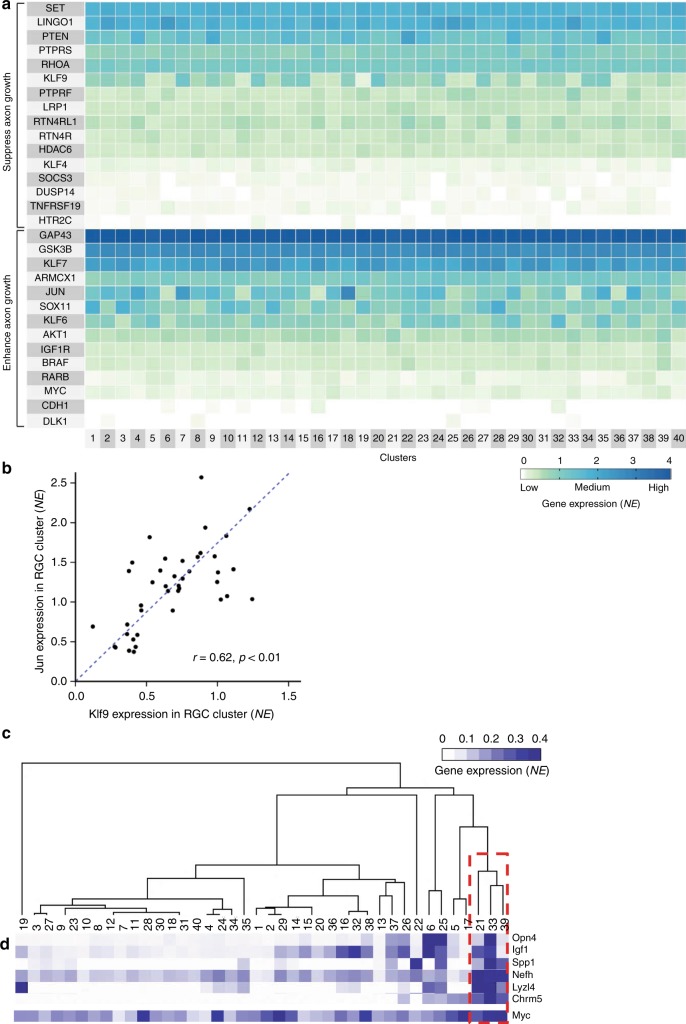
RGC subtypes enriched for axon growth-regulating genes. **a** Heatmap of genes known to regulate axon growth and regeneration shows differential expression between RGC subtypes. **b** Correlation between enrichment of Klf9 and Jun genes expression in different RGC subtypes, *r* = 0.62, *p* < 0.01 (Pearson, 2-tailed). **c** Heatmap of genes enriched in melanopsin-expressing ipRGCs, and a dendrogram showing clustering of putative αRGC subtypes (red box). **d** Myc gene is highly expressed in putative αRGC subtypes

Because RGCs differ in capacity to regenerate injured axons in response to treatments^48,50,51^, we asked whether some subtypes are enriched for axon growth suppressing or promoting genes. We found a number of axon growth-regulating genes differentially expressed between different subtypes (Fig. 9a). We recently showed that anti-Klf9 shRNA stimulates regeneration of injured axons from a subset of RGCs^52^. Thus, we tested whether Klf9 expression differs between RGC subtypes. We found that Klf9 was enriched the most in ISP 10 (4 out of 5 subtypes: 7, 20, 27, 34), and to a lesser extent in ISP 9 (4 out of 5 subtypes: 9, 14, 15, 21), ISP 1 (22 and 23), ISP 6 (4 out of 5 subtypes: 4, 18, 35, 29), and ISP 8 (3 out of 5 subtypes: 2, 12, 24). Thus, a modest number of RGCs that responded to the anti-Klf9 shRNA treatment may be due to that Klf9 suppresses regeneration of only some subtypes. Another axon regeneration-regulating gene, Jun^53,54^, showed a similar pattern of subtypes enrichment as Klf9, and we found significant correlation between Klf9 and Jun expression in different subtypes (Fig. 9a, b).

The αRGC subtype regenerates injured axons in response to inhibition of Pten^48,50^. The αRGC is an M4 subtype of the ipRGCs, which are labeled by Opn4^16^ and include subtypes M1-M4^50^. M4/αRGCs express less Opn4 than M1-M3 and co-expresses axon growth-promoting Spp1 and Igf1^48^. In our data set, subtypes 6, 25, 26, 33, and 37 expressed Opn4 and constituted ISP 2 (Fig. 8); all five also expressed Igf1 and Eomes^17^ but only 25, 33, and 37 also co-expressed Spp1 (Figs. 3e and 9c). Subtypes 5, 15, 21, 38, and 39 (that belong to ISPs 7-9; Fig. 8) expressed Opn4 weekly and co-expressed Igf1, but only subtypes 21 and 39 also co-expressed Spp1, and only subtype 5 also co-expressed Eomes. Thus, all Opn4 + RGC subtypes co-express Igf1 but only some also co-express Spp1 or Eomes. To explore which of these Opn4+ RGC subtypes are αRGCs, we analyzed for enrichment of the Nefh mRNA, which encodes Neurofilament-H containing a non-phosphorylated epitope recognized by an SMI-32 antibody that labels αRGCs^48,50^. We found that Nefh was significantly enriched only in subtypes 33, 39, and 21 (Fig. 9c), suggesting that these are αRGC subtypes (see Supplementary Discussion). We also found that axon regeneration-promoting Myc^55^ was associated with αRGCs (Fig. 9d), and that Chrm5 and Lyzl4 were co-enriched in the predicted αRGC subtypes (Fig. 9c); see Supplementary Discussion.

### RGC subtypes gene browser

Finally, we developed RGC Subtypes Gene Browser, (https://health.uconn.edu/neuroregeneration-lab/rgc-subtypes-gene-browser) that provides a platform for analyzing and comparing gene expression profiles in the RGC subtypes. The browser offers three types of analyses for any gene in every RGC subtype: Means and SEM for the left and right eyes^56^, Violin Plot of cell density for different gene expression levels^38^, and Notched Box Plot for comparing medians using the Tukey Box-and-Whisker plot with whiskers set to 1.5 times the interquartile range (IQR)^57–59^.

## Discussion

Thirty subtypes of RGCs have been established to date based on differences in morphology, localization, function, and susceptibility to degeneration^9,15^ (see Supplementary Discussion). We classified RGCs into 40 subtypes based on their transcriptome profiles, and identified genes uniquely enriched in these subtypes. Because we used RGCs from a pre-eye-opening age, our data suggests that initial subtype specification does not require visual stimulation^18,32^, and that changes in gene expression during further maturation^33^ may be fine-tuning or further subdividing already specified subtypes, as well as affecting RGC housekeeping functions, such as the inactivation of intrinsic axon growth capacity^34–36^. Remarkably, 40 subtypes we found matches the number of RGC categories that were recently classified based on electrophysiological properties^37^. Future studies are needed to determine how the molecular differences between subtypes underlie these electrophysiological functions, and also to investigate whether they differ in morphology, retinal spatial distribution, target cell connectivity, and associated visual parameters.

Most of the RGC markers known to date were enriched in more than one RGC subtype, and may represent intermediate subpopulations of RGCs. Five of the known markers (Jam2, NPY, Pde1a, Trhr, and Gna14; see Supplementary Discussion) were specific to the subtypes we predicted, and we also validated three more subtype-specific markers (Runx1, Fst, and Zic1). Experimental evidence for the validity of 8 out of 40 predicted subtype markers, along with statistical support for the remaining predicted subtype-specific markers, highlight the importance of validating the rest of the markers, as well as multiplexing different markers in situ, in future studies. We also predicted the putative midget and α RGC subtypes and markers (see Supplementary Discussion), and defined subpopulations and specific markers of ooDSGCs and ipRGCs.

We predicted TFs that are uniquely enriched in RGC subtypes individually or in combinations (and validated two of them, Runx1 and Zic1). It would be important to investigate whether these TFs cooperate with each other in specifying respective subtypes during development. For example, to test whether they bind to the same DNA regulatory elements in RGCs. Because enhancers show significantly more cell type and subtype specificity than promoters in regulating gene expression^60–62^, promoter analyses for the TF binding sites would not be sufficient to address this question, but it should be addressed when future studies will reveal the profile of enhancers active specifically in RGCs. We showed hierarchical scheme of RGC subtypes diversification, from a population to subpopulations and then to subtypes, that may reflect functional specializations, some of which are already known (e.g., ooDSGC for the ISP 1, and ipRGC for the ISP 2), and the remaining ISPs need to be linked to already known or yet to be uncovered functions. Hierarchy and order of TFs expression in combinations was shown to guide differentiation of different cell fates^63–65^, and phylogenetic tree-like analyses were linked to differentiation pathway^66,67^. Thus, hierarchy we found may also reflect developmental specification, as we have shown unique combinations of TFs in the ISPs (some of which are already known to be necessary for differentiation of certain RGC subtypes as discussed above), which may pre-differentiate them for further specification into subtypes by subtype-specific TFs.

We also showed that the brain laterality may be to some extent predetermined intrinsically at the level of subtype specification, rather than shaped by experience alone. Although overall left and right eye RGC subtypes transcriptomes mirror each other, a few of RGC subtypes were overrepresented in one eye, suggesting that some eye functions may be predominant in one eye^68^, analogous to speech-related functions enriched in the Broca and Wernicke areas of one hemisphere. While intrinsic molecular underpinnings of brain asymmetry remain elusive, a number of single-gene models have been suggested^69^. Thus, subtype-specific marker Zic1 enriched in the right eye RGCs may be a molecular marker of intrinsically-specified laterality. We also found that in both eyes Zic1 localized predominantly in the ventrotemporamedial retina. Whether Zic1 plays a role in right eye-predominant putative function remains to be investigated.

Our analyses are consistent with the hypothesis that RGCs differ in response to the treatments that promote axon regeneration. For example, we identified RGC subtypes co-enriched for Klf9 and Jun, suggesting that although Klf9 is a suppressor and Jun is an enhancer of axon regeneration, a balance between them may regulate axon growth in respective subtypes. We also identified subtypes that match the markers of αRGCs, that respond to an anti-Pten shRNA for regenerating a subset of injured axons^48^ (see Supplementary Discussion). Because Klf9 was highly expressed only in one out of the 3 predicted αRGC subtypes, bulk of the anti-Klf9 shRNA effect on axon regeneration is expected to be through non-αRGCs, and thus a co-treatment with anti-Klf9 and anti-Pten shRNAs may lead to a more robust regeneration than each alone.

This data set is a resource for studying gene networks and pathways in RGC subtypes, including subtypes’ varying response to neuroprotective/regenerative treatments. The RGC Subtypes Gene Browser provides a platform for such analyses, to assist scientific community in the investigation of the molecular and physiological differences between RGC subtypes.

## Methods

### Animals

All animal procedures were approved by the University of Connecticut Institutional Animal Care and Use Committee and by the Institutional Biosafety Committee at the University of Connecticut, and performed in accordance with the ARVO Statement for the Use of Animals in Ophthalmic and Visual Research. C57BL/6 J mice were obtained from Charles River Laboratories, Inc.

### RGC purification, culture, immunostaining, and FISH

RGCs were purified from both sexes of postnatal day 5 mice eyes single-cell suspension (separately for the left and right eyes) by immunopanning for Thy1 (CD90, MCA02R, Serotec) after depletion of macrophages (using anti-mouse macrophage antibody, AIA31240, Accurate Chemical) and washing off the nonadherent cells, following our established protocol^19^. To confirm purity and survivability of RGCs, cells were plated and cultured in defined growth medium^19,21,24^, and immunostained after 12 h with an RGC marker RBPMS (1832-RBPMS, PhosphoSolutions), neuronal marker Tuj1 (MMS-435P, BioLegend), and DAPI (Thermo Fisher Scientific). As shown in Fig. 1a, all the cells (DAPI+) had healthy morphology (DAPI+/Tuj1+) and were RGCs (DAPI+/Tuj1+/RBPS+), and no apoptotic or stress markers were found amongst the genes enriched in individual clusters (Fig. 4), thus showing that there were no apparent biases in subtype survivability due to prep/processing. For validating expression of Fst, immunostaining was performed similarly, except Fst (1:3000; AB203131, Abcam) instead of Tuj1 antibody was used. AlexaFluor fluorophore-conjugated secondary antibodies (Thermo Fisher Scientific) were used for fluorescent microscopy (with AxioObserver.Z1, Zeiss). For fluorescent in situ hybridization (FISH), cells were fixed at 12 h as for immunostaining, and then probed by single-molecule sensitivity ViewRNA Cell Plus Assay kit (Thermo Fisher Scientific) with the probes for Runx1 and RBPMS and co-stained with DAPI. In an experiment where FISH for Runx1 was followed by immunostaining for Fst, representative fields of view with the cells were acquired after FISH, and then manually cross-referenced to images from subsequent immunostaining for Fst (channels were merged using Piant.Net Layers tools). Two rounds of imaging were necessary, because even post-fixing after FISH did not prevent the loss of Runx1 signal following Fst immunostaining. AlexaFluor 647 was used for Fst in this experiment (shown in green pseudocolor), because AlexaFluor 594 we used in the initial characterization of Fst+ RGCs was too close in wavelength to the 546 probe for Runx1.

### Immunostaining of retinal sections

Eyes from mature mice were dissected and fixed for 2 h in 4% paraformaldehyde after puncturing the cornea, washed in PBS, incubated overnight in 30% sucrose at 4 °C, and cryosectioned (14 µm). Right after the eye was dissected-out, a needle was inserted through the cornea on a temporal side of the eye in dorsal to ventral direction, with the needle hub at the dorsal side. After fixation the needle was removed and the eyes were embedded in OCT mold with the eye identity (left or right) and orientation (ventral facing one side and dorsal the opposite side in one direction, and in perpendicular direction temporal and nasal orientation facing opposite sides) marked on the outside of the mold. Slides were marked accordingly, to preserve the information regarding the orientation of cryosections. The cryosections were immunostained for Zic1 (AB134951, Abcam), an RGC marker RBPMS (1832-RBPMS, PhosphoSolutions), and DAPI (Thermo Fisher Scientific). Alexa fluorophore-conjugated secondary antibodies (Thermo Fisher Scientific) were used for fluorescent microscopy (with AxioObserver.Z1, Zeiss). RBPMS+/Zic1+ and RBPMS+/Zic1− RGCs were quantified from nasal, medial, and temporal retinal regions in sections from dorsal and ventral retina, in left and right eyes from four animals each.

### Single-cell RNA-seq

RGCs were purified (as described above) from 8 P5 pups of both sexes in parallel, separately from the right and left eyes, resuspended in DPBS with 0.04% BSA, and immediately processed as follows. RGC count and viability were determined using trypan blue on a Countess FL II, and 6000 cells from each eye were loaded in parallel for capture onto the Chromium System using the v2 single-cell reagent kit (10 × Genomics). Following capture and lysis, cDNA was synthesized and amplified (12 cycles) as per manufacturer’s protocol (10 × Genomics). The amplified cDNA from each channel of the Chromium System was used to construct an Illumina sequencing library and sequenced on HiSeq 4000 with 150 cycle sequencing. Illumina basecall files (*.bcl) were converted to FASTQs using CellRanger v1.3, which uses bcl2fastq v2.17.1.14. FASTQ files were then aligned to mm10 mouse reference genome and transcriptome using the CellRanger v1.3 software pipeline with default parameters (as described in ref. ^22^), which demultiplexes the samples and generates a gene versus cell expression matrix based on the barcodes and assigned unique molecular identifiers (UMIs) that enable determining from which individual cell RNA molecule originated. Because high coverage depth of 5000 genes per cell, on average, was achieved, a sub-peak of cells below 3000 genes per cell (Fig. 1b) was excluded due to poor coverage, in order to improve reliably of downstream analysis. Gene expression was normalized using CellView software^23^. Briefly, the number of gene transcripts per cell is multiplied by the median of transcripts across all the cells, and then log2-transformed (following an addition of +1 pseudocount to prevent log error where transcripts count is 0, i.e., log2(0 + 1) = 0), resulting in NE values. For clustering, genes were selected based on normalized dispersion analysis. Sex-specific genes (male Eif2s3y and Ddx3y, and female Xist) were excluded from the clustering steps but were retained in the data set for downstream analysis after clustering. Two-round dimensionality reduction was performed using CellRanger^22^ and CellView^23^ pipeline: first, dimensionality was reduced with the 1000 most over-dispersed (i.e., variance/mean NE) genes using Barnes-Hut t-SNE with default parameters, and cell clusters were determined using DBSCAN (eps = 5.0, minpts = 15); next, the clusters were checked for RGC (Fig. 1e and Supplementary Figure. 2C) and non-RGC cell-type markers, and the few outlier non-RGC clusters comprised of only 253 cells in total (representing only a minor 3.9% contamination with non-RGCs) were excluded from the next round of clustering. Total of 6225 RGCs (2493 from left and 3732 from right eyes) were used in this final clustering step. Clusters were visualized (Fig. 1d) using the t-SNE 2D graph with the 3rd dimension color-coded. The data set is available through the NCBI GEO accession number GSE115404 Series.

### Heatmaps and dendrograms

For Fig. 1f, we preformed unsupervised *k*-means clustering to generate the heatmap along with hierarchical clustering for the dendrogram, using the R package Superheat^70^ with maximum distance computation and the default linkage method. The heatmaps in Figs. 3e and 9a were also generated with Superheat, but the order of rows was specified based on the more prevalent markers shown at the upper rows, and the columns were specified in an increasing order from cluster 1 through 40. For Fig. 4 heatmap, expression values for each gene across different clusters were normalized for each row prior to plotting by the centered columns scaling R function with the *z*-scores, and the columns were then specified in an increasing order from cluster 1 through 40. For Fig. 7a circular phylogenetic tree-style diagram of RGC clusters, we preformed unsupervised hierarchical clustering with the Ward’s method, using the R package Ape^57,71^ with maximum distance computation. For Fig. 9c and Supplementary Figure. 3, we preformed clustering and visualization using Gene Cluster 3.0 and Java Treeview 1.1.6r4^72,73^, with genes normalized, uncentered Pearson correlation, and average linkage, to generate heatmaps and dendrograms.

### Bulk and single-cell RNA-seqs correlation analysis

Gene expression from all individual cells in the scRNA-seq data set was averaged, and then compared through 2-tailed Pearson correlation analysis with bulk RGC mRNA-seq, which we generated previously using polyA-selected RNA and paired reads sequenced 100 bp from each end^3^. For this analysis, genes expressed < 1 FPKM in the bulk RGC RNA-seq were excluded due to noise, as we previously reported^3^. Fit line in the scatterplot was plotted using LOESS.

### Analysis of clusters enrichment per eye

Proportions of cells per cluster per eye were analyzed using SPSS Custom Tables, Comparisons of Column Proportions (eyes in columns, clusters in rows). Pearson *χ*^2^ test with df 39 was significant at *p* < 0.001, and 2-sided *z*-score-based tests of column proportions, with pairwise comparisons adjusted using the Bonferroni correction, was significant at *p* < 0.001 for the cluster 34 right eye cells in larger proportion, and also at a higher threshold of *p* < 0.01 for the clusters 19 and 37 left eye cells in larger proportion.

### Thresholds for signature genes enriched in RGC subtypes

All genes reported to be enriched uniquely per cluster were expressed in a respective subtype 1.8-fold more compared to any other cluster at *p*-value ≤ 0.05. Threshold of 1.8-fold was selected because it predicted at least one uniquely enriched gene marker for every single cluster. In addition, a gene had to be expressed >0.05 NE to be considered enriched. The *p*-value was calculated for up to top 5 markers per subtype, shown in Fig. 4. The *p*-values were calculated using R software by independent samples *t*-test (2-tailed), as well as by a nonparametric independent samples Mann–Whitney *U* test, for gene NE between all the cells (*n* in sample 1) comprising respective cluster and all the cells (*n* in sample 2) in another cluster. Then, if *p*-value was ≤ 0.05 in each of the 39 comparisons (between respective cluster and every other cluster) using both tests, a gene was considered significantly enriched and its name shown in black font in Fig. 4. If *p* ≤ 0.05 by the *t*-test and *p* ≤ 0.1 by the Mann–Whitney *U* test, the gene name is shown in green font. If *p* ≤ 0.05 by the *t*-test but *p* > 0.1 by the Mann–Whitney *U* test, the gene name is shown in red font. An exception to these thresholds was made only for cluster 40, because it had the smallest number of cells than any other cluster, which resulted in *p* > 0.1 by either test for the top 2 out of 3 most fold enriched genes, indicated by asterisk in Fig. 4. Supplementary Data 1 also includes other genes that met only the first two criteria.

### Combinatorial analysis of TFs enrichment in RGC subtypes and ISPs

TFs enriched as a unique combination (but not individually) in single clusters were identified by up to five-way combinatorial analysis using MATLAB software. TF that is expressed in a cluster > 0.5 NE and >twofold compared to its mean NE of all clusters was assigned a value of 1 in a TF × Clusters binary matrix. A value of 0 was assigned for other clusters in which the TF did not meet these criteria. Thus, each row representing a TF had at least one “0” and at least one “1” that corresponded to clusters (columns). Next, the *nchoosek* function was used to generate a virtual matrix of all TF combinations (up to five ways), and within each combination multiplication eliminated combinations containing 0 in at least one cluster. This created an intermediate virtual binary matrix, where each remaining combination (row) was assigned a value of 1 in clusters (columns). Then, only combinations whose sum per row was = 1 were retained as unique cluster signatures (i.e., if sum > 1 then the combination also co-occurs in another cluster). To further filter for even more enriched combinations, we widened the gap between an enrichment of TFs combination unique to an individual cluster and the next best cluster match (i.e., where such combination may match but at a lower fold enrichment). To widen this gap, the pipeline was re-run as above, with an exception of that in the initial filtering a TF had to be only >1.5-fold more than its mean NE of all clusters. With this lower threshold, more TF combinations passed into an intermediate matrix, which in turn resulted in more combinations being eliminated due to their co-occurrence in more than one cluster but also some new combinations with lower enrichment passing. Thus, we then cross-referenced the outputted combinations to those from the first run (that used a twofold threshold), and retained only those that matched between these two lists (i.e., thereby widening the gap between an enrichment of TFs combination unique to an individual cluster and the next best cluster match). Statistical interaction of the predicted TFs enrichment as a combination (i.e., interaction of two continuous dependent variables) in respective clusters (i.e., an independent variable) relative to every other cluster was analyzed by Repeated Measures ANOVA with Tukey’s HSD post hoc for pairwise comparisons (SPSS), and was significant at *p* ≤ 0.05 for almost all RGC subtypes (a few exceptions are explained in Table 1 legend), with sphericity assumed. For comparison, TFs’ statistical interaction in all other clusters compared to each other (excluding the cluster in which the combination was predicted to be enriched) was analyzed similarly, and it was not significant for any subtype. Combinatorial analysis of the TFs combinations enriched in the ISPs were performed similarly, except that the clusters comprising an ISP were first merged into one. Analysis of single TFs uniquely enriched in ISPs was also performed on clusters merged into ISPs. Subsequently, an output was also manually checked to confirm that each predicted TF is in fact enriched in every cluster of its respective ISP. This manual filtering step was needed because when comparing the averages of the merged ISP clusters, a contribution of TF expression in each individual cluster is not considered. This step led to exclusion of some TFs and combinations in which a predicted TF was not enriched in one of the ISP clusters. The remaining combinations are listed italicized on the side of Fig. 7a.

### Design of the website and online tools

The RGC Subtypes Gene Browser was designed using R and ShinyApps with R-markdown language^57^. Means and SEM functions were adapted from ggplot2 R software package for data visualization^56^. Boxplot and violin functions were adapted from R statistical software packages, box plot^57^ and simple.violinplot^38^.

### Data availability

The data set we generated for these studies is available through the NCBI GEO accession GSE115404 (https://www.ncbi.nlm.nih.gov/geo/query/acc.cgi?acc = GSE115404). Access to the data set is also available through a user-friendly web application, RGC Subtypes Gene Browser, https://health.uconn.edu/neuroregeneration-lab/rgc-subtypes-gene-browser.

## Electronic supplementary material

Supplementary Information

Description of Additional Supplementary Files

Supplementary Data 1

Supplementary Data 2
