# Author Correction: Single cell transcriptome profiling of retinal ganglion cells identifies cellular subtypes

**DOI:** 10.1038/s41467-018-05792-3

**Published:** 2018-08-07

**Authors:** Bruce A. Rheaume, Amyeo Jereen, Mohan Bolisetty, Muhammad S. Sajid, Yue Yang, Kathleen Renna, Lili Sun, Paul Robson, Ephraim F. Trakhtenberg

**Affiliations:** 1 Department of Neuroscience, University of Connecticut School of Medicine, 263 Farmington Ave, Farmington, CT 06030 USA; 2 The Jackson Laboratory for Genomic Medicine, Farmington, CT 06032 USA; 3 Institute for Systems Genomics and Department of Genetics & Genome Sciences, University of Connecticut School of Medicine, Farmington, CT 06032 USA

Correction to: *Nature Communications*; 10.1038/s41467-018-05134-3; published online 17 July 2018

The original version of the Supplementary Information file associated with this Article contained an error in Supplementary Fig. 2. In panel c, the graph was inadvertently replaced with a duplicate of the graph in panel a. The error has now been fixed and the corrected version Supplementary Information PDF is available to download from the HTML version of the Article.

