# Supplementary material for: Single cell transcriptome profiling of retinal ganglion cells identifies cellular subtypes
Source: Nat Commun. 2018 Jul 17;9:2759. doi: 10.1038/s41467-018-05134-3 (PMC6050223; doi:10.1038/s41467-018-05134-3)
Supplement: Supplementary file 1 — Supplementary Information [file 41467_2018_5134_MOESM1_ESM.pdf]

Single cell transcriptome profiling of retinal ganglion cells identifies cellular subtypes
Rheaume, et al.

Supplementary Information

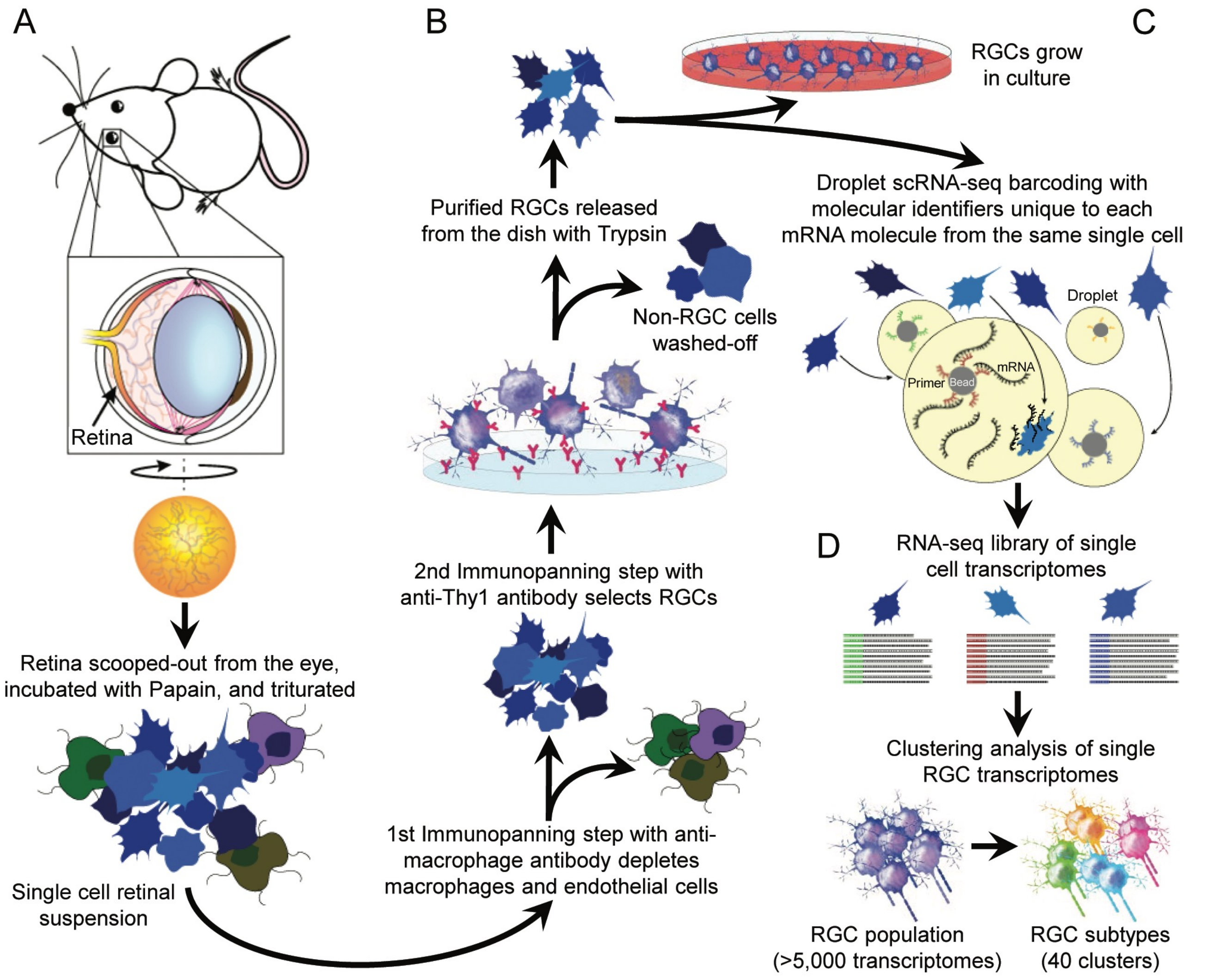

Supplementary Figure 1 | Schematic of RGC purification and single cell RNA-seq. (**A**) Left and right eye retinas are dissected and dissociated into single cells separately in parallel. (**B**) The retinas and RGCs are purified by immunopanning. Purified RGCs are cultured or immediately processed with droplet-based scRNA-seq. (**C**) In the 10x Genomics Chromium droplet-based scRNA-seq method, single cells are individually encapsulated into a droplet along with barcoded primers bound to a bead. Cells are lysed within droplets, and the mRNAs are bound to oligo dT barcoded primers on the beads. This procedure enables the barcodes to provide the information about the cell that a cDNA has originated from and the specific mRNA molecule, using a unique molecular identifier (UMI). UMIs also enable a digital mRNA count that eliminates the amplification bias. (**D**) An RNA-seq library of single cell transcriptomes is mapped (mm10) and normalized gene expression profiles are generated using the CellRanger software, which is a part of the droplet-based scRNA-seq 10x Genomics Chromium platform. A subsequent clustering analysis of single cell transcriptomes segregates the RGC population into subtypes.

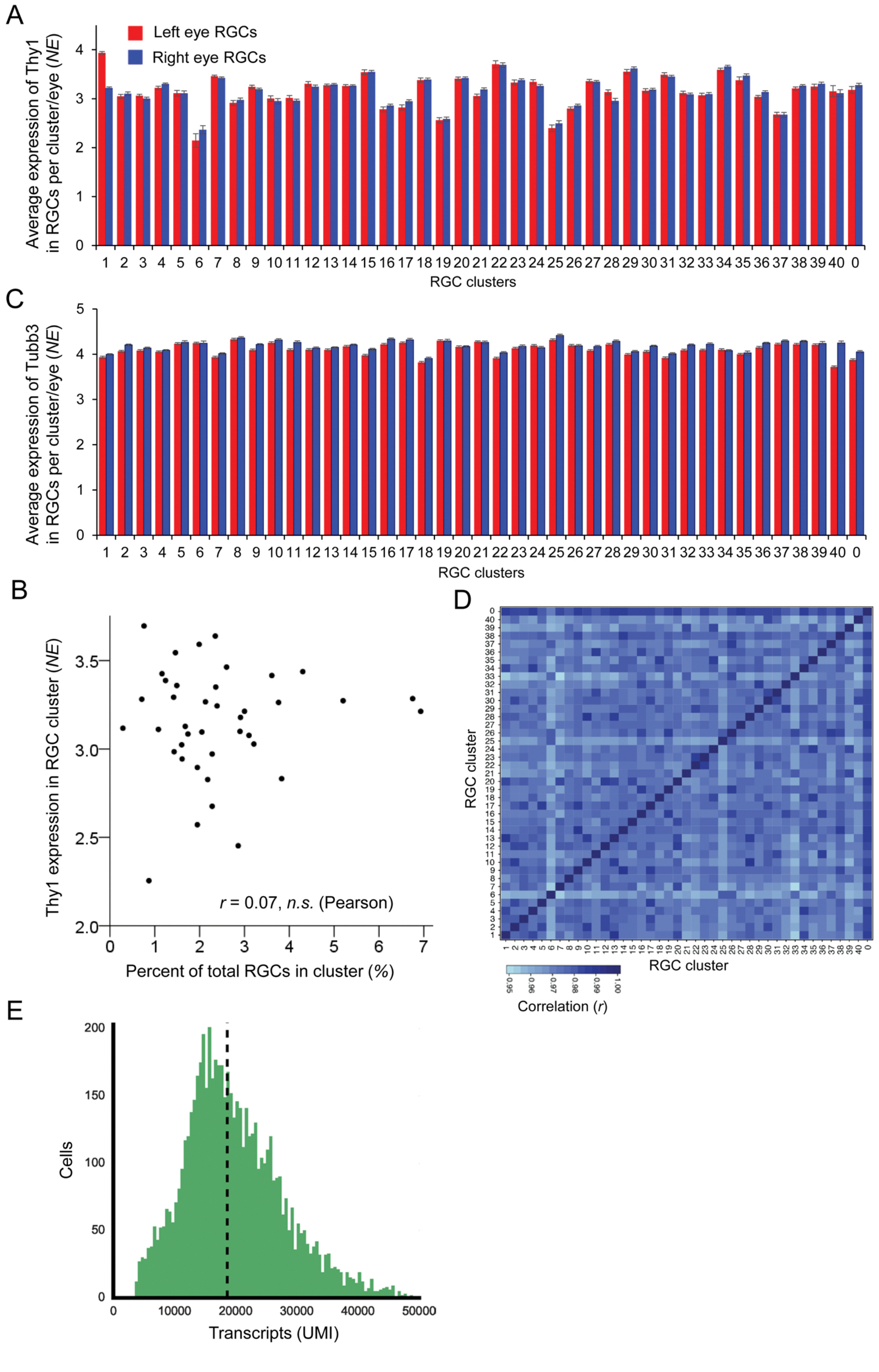

Supplementary Figure 2 | Pan RGC marker expression, cluster correlation, and depth of coverage. (**A**) Although high in all RGC clusters, Thy1 expression levels vary amongst the clusters but in similar proportions between left and right eyes across all clusters. Mean ± SEM shown. **(B**) A scatterplot shows no correlation between the level of Thy1 expression in cluster and percent of RGCs comprising the cluster (r = 0.07, *n.s.*, Pearson). (**C**) Tubb3 is expressed highly and similarly in all RGC clusters, and also in a similar proportion between left and right eyes across all the clusters. Mean ± SEM shown. (**D**) A correlation matrix of RGC cluster gene expression profiles ($r > 0.9$ by Pearson 2-tailed, all correlations, $p < 0.01$). (**E**) Coverage depth of unique transcripts per cell based on UMI counts.

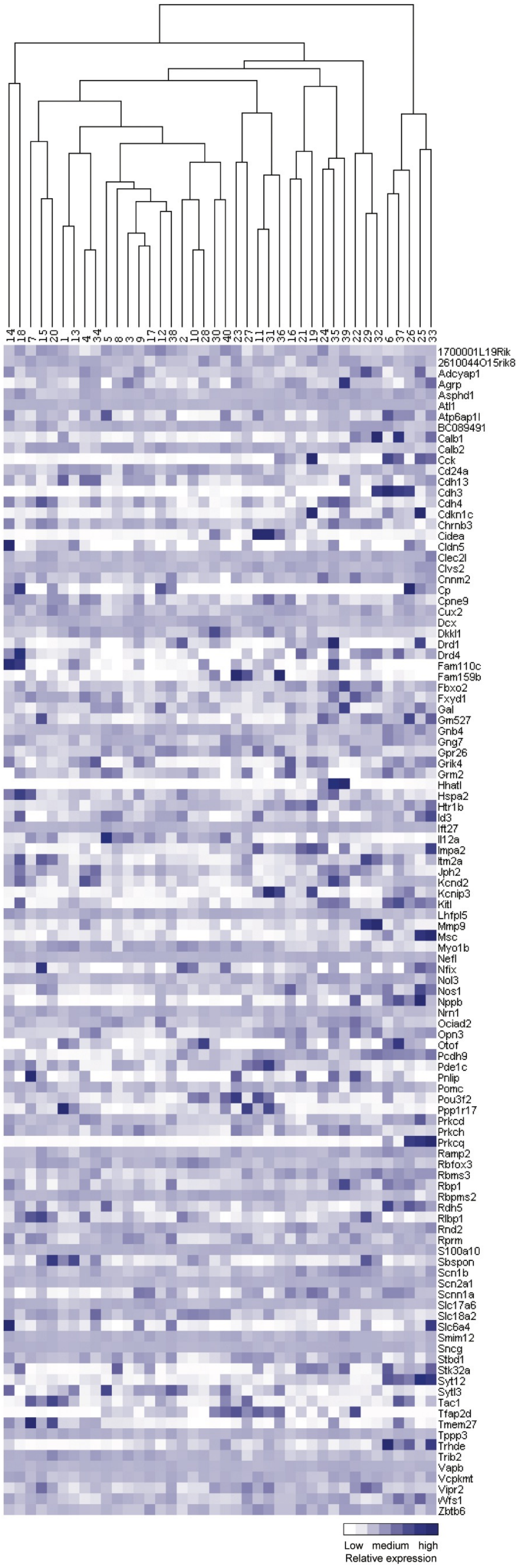

Supplementary Figure 3 | Heatmap of RGC subset markers. Genes shown to be expressed in subsets of RGCs in prior screens, which we have found enriched in more than one RGC cluster.

Supplementary Table 1 | Percent of RGCs per eye. Percent of RGCs in each cluster per eye respective to the total number of RGCs in that eye (left, *n* = 2,493; right, *n* = 3,732). Data is sorted based on the ratio of the percent of RGCs in right eye over those in the left per cluster.

Cluster	% Right RGCs	% Left RGCs	Ratio (right/left)
40	0.46	0.12	3.79
34	3.05	1.64	1.86
32	2.47	1.64	1.50
18	1.47	1.00	1.47
31	3.08	2.13	1.45
30	3.30	2.53	1.30
13	7.48	6.02	1.24
22	0.83	0.68	1.22
12	2.33	1.93	1.21
4	5.63	4.77	1.18
36	3.08	2.73	1.13
38	2.49	2.29	1.09
24	1.47	1.36	1.08
10	2.36	2.21	1.07
28	1.63	1.56	1.04
14	3.83	3.69	1.04
27	2.38	2.33	1.03
29	2.01	1.97	1.02
15	1.47	1.44	1.02
23	1.50	1.48	1.01

Cluster	% Right RGCs	% Left RGCs	Ratio (right/left)
7	4.31	4.29	1.01
17	1.93	1.97	0.98
33	1.71	1.76	0.97
39	0.70	0.72	0.96
20	3.54	3.69	0.96
16	2.12	2.25	0.94
11	1.37	1.48	0.92
9	2.84	3.17	0.90
1	6.51	7.34	0.89
3	2.92	3.49	0.84
25	2.60	3.13	0.83
5	0.96	1.20	0.80
26	3.40	4.25	0.80
21	1.47	1.89	0.78
2	2.68	3.53	0.76
35	0.96	1.36	0.71
6	0.70	1.04	0.67
37	1.71	2.85	0.60
8	1.21	2.01	0.60
19	1.45	2.45	0.59

Supplementary Table 2 | RGC subtype markers that matched prior screens. (*Top section*) 20 of the markers we found enriched in specific clusters were also shown to be expressed in subsets of RGCs in previous screens[1-3]. (*Lower section*) Five TFs from the TF combinations, which we found uniquely enriched (as a combination but not individually) in some of the clusters or ISPs, were also shown in previous screens to be expressed in subsets of RGCs; some of the others that are known to label subsets of RGCs are shown in Fig. 3E.

Gene	Publication
Genes enriched in individual RGC clusters	
Runx1	Rousso et al., 2016[3]
Syt6	Martersteck et al., 2017[1]
Pdzk1ip1	Martersteck et al., 2017[1]
Chrnb4	Martersteck et al., 2017[1]
Crh	Martersteck et al., 2017[1]
Neto1	Martersteck et al., 2017[1]
Cyp1b1	Siegert et al., 2012[2]
Dcn	Siegert et al., 2012[2]
Gpr101	Siegert et al., 2012[2]
Hes1	Siegert et al., 2012[2]
Plpp3	Siegert et al., 2012[2]
Serpinb1b	Siegert et al., 2012[2]
Prph	Siegert et al., 2012[2]
Tmem255b	Siegert et al., 2012[2]
Prss34	Siegert et al., 2012[2]
1700011I03Rik	Siegert et al., 2012[2]
Ctxn3	Siegert et al., 2012[2]
Car4	Siegert et al., 2012[2]
Pllp	Siegert et al., 2012[2]
Slc16a9	Siegert et al., 2012[2]
TFs enriched in subsets of RGCs	
Etv1	Rousso et al., 2016[3]
Bcl11b	Rousso et al., 2016[3]
Zbtb16	Rousso et al., 2016[3]
Pou6f2	Siegert et al., 2012[2]
Irx6	Siegert et al., 2012[2]

SUPPLEMENTARY DISCUSSION

The dataset we generated enabled us to tackle some of the general questions in cell type and subtype biology, as well as to determine the transcriptomes of previously identified RGC subtypes. Below we provide additional discussion of our results, as they relate to these questions.

Primary literature on RGC subsets and subtypes markers

- RGC subsets and subtypes established to date based on differences in morphology, localization, function, susceptibility to degeneration, and regenerative caapcity[1,3-45].
- Previously described RGC subtype-specific markers and RGC subsets labeled in transgenic mouse lines[1,3-6,9-45].
- Pan-markers that label all or many RGC subtypes[1,9-13,18,22-29,46].
- RGC subtype or subset-specific markers[3,6,19,20,30-45].
- Five previously reported RGC markers we found to be subtype-specific[31,32,35,42,43].

Thresholds that distinguish between cell types and subtypes

To what extent do cells need to be similar to each other to be a member of a cell type, and what extent of variability within a cell type may be sufficient for segregation into subtypes? We found that a very narrow variability range in the transcriptome is sufficient for distinguishing between RGC subtypes on a molecular level, while all the subtypes comprising a cell type correlate with each other > 0.9 (*r*, Pearson). This is in contrast to much larger differences between RGC and other cell types[47], although different cell types within the same tissue may be more closely related[48]. Classification of more cell types and their subtypes in the future will help determine whether there are indeed generalizable thresholds in similarity between cells that molecularly distinguish between cell types and subtypes, and whether tissue, lineage, or other property is weighing more on setting such thresholds. Future studies are also needed to investigate how such molecular subtyping thresholds may underlie thresholds from other criteria (e.g., morphological, connectional, electrophysiological).

Global properties of cell type-maintaining and subtype-specific genes and TFs

We found that more TFs and genes are involved in differentiating between subtypes than in maintaining a cell type, however, they are expressed highly only in some subtypes and consequently present as low expressed when averaged across all RGCs at the cell type level. On the other hand, bulk of the transcriptome (i.e., total RNA molecules per cell) is involved in maintaining an RGC as a cell type, even though it implicates fewer distinct genes, which is consistent with the hypothesis that fine-tuning a cell type into a subtype would not require as much of the transcriptome as maintenance of a cell type itself. Our results are also consistent with the expectation that the TFs are expressed at lower levels than the genes they regulate[49], however, our data suggests that this difference is more pronounced in cell type-maintaining rather than subtype-specifying TF-gene relationship and that it may not exist at very high levels of expression. As various cell types differ in the proportion of highly expressed genes[47], those highly expressed genes may be involved in defining respective cell types rather than subtypes within them. Increasing the database of cell type and subtype transcriptome profiles will allow investigating whether these are generalizable principles across certain tissues.

Subtype enrichment and its perceived importance for a cell type as whole

Our data suggests that the proportion of cells comprising a neuronal subtype may not be associated with its perceived functional importance at the cell type level. For example, although ipRGCs that are involved primarily in entraining circadian rhythms may be perceived as more dispensable than, for example, direction-selective or ON-OFF RGC subtypes, we did not find this subpopulation to be underrepresented nor any subtype to be substantially overrepresented.

Prediction of the putative midget RGC subtypes

Midget RGCs are the smallest and most abundant RGC subtype in primates[50], but their specific gene markers are unknown to date[3], and RGCs reminiscent of primate midget RGCs were identified in mouse retina only electrophysiologically[51]. As the global size of the transcriptome is related to cell size[47,52], the RGC subtypes 1 and 13, that have the highest number of cells expressing the fewest genes and are also the most abundant subtypes, may represent midget RGCs. Furthermore, the association of RGC subtypes 1 and 13 under the same intermediate subpopulation (ISP 4, **Fig. 7A**), supports the hypothesis that these subtypes are closely related, and may represent different classes of midget RGCs.

Prediction of the αRGC subtypes and markers

Because αRGCs have the largest soma amongst the RGCs[6] and the transcriptome and cell sizes are associated[47,52], it is possible that subtypes 33 and 39, which we found have the largest size transcriptome (**Fig. 3A**) and also co-express Opn4, Igf1, and Spp1, are the αRGCs. Particularly subtype 39, which expresses lower level of Opn4 mRNA compared to other Opn4+ subtypes. Since subtype 21 also expresses Opn4 less than the other Opn4+ RGC subtypes and co-expresses Igf1 and Spp1, it may also be an αRGC, although its transcriptome size is only somewhat higher than that of an average RGC subtype (**Fig. 3A**). Our finding that Nefh (an αRGC marker[6,53]) is significantly enriched only in subtypes 33, 39, and 21 (**Fig. 9C**), supports the hypothesis that these are αRGC subtypes.

αRGC Myc activity may enable anti-Pten shRNA to promote axon regeneration

αRGCs respond to an anti-Pten shRNA for regenerating a subset of injured axons[6], and we found that axon regeneration-promoting Myc[54] was associated the RGC subtypes which we predicted to be αRGCs (**Fig. 9D**). This observation supports the hypothesis that αRGC Myc pathway may cooperate with anti-Pten shRNA to lead to axon regeneration. This is consistent with a recent report that, upregulating Myc expression in RGCs beyond endogenous levels acts synergistically with the inhibition of Pten in promoting more robust regeneration (presumably in αRGC) than either treatment alone[54].

αRGCs are predicted to respond to the cholinergic amacrine cells through Chrm5 receptor

We performed gene expression pattern analysis, and found that Chrm5 and Lyzl4 were co-enriched in the predicted αRGC subtypes (**Fig. 9C**). Chrm5 is a muscarinic acetylcholine receptor. A subtype of cholinergic amacrine cells that synapse on RGCs has been characterized[30,55], and nicotinic acetylcholine receptors nave been found in subsets of RGCs[56,57]. Thus raising the hypothesis that αRGCs may uniquely respond to cholinergic amacrine cells through a muscarinic acetylcholine receptor, Chrm5.

SUPPLEMENTARY REFERENCES

1 Martersteck, E. M. *et al.* Diverse Central Projection Patterns of Retinal Ganglion Cells. *Cell Rep* **18**, 2058-2072, doi:10.1016/j.celrep.2017.01.075 (2017).

2 Siegert, S. *et al.* Transcriptional code and disease map for adult retinal cell types. *Nat Neurosci* **15**, 487-495, S481-482, doi:10.1038/nn.3032 (2012).

3 Rousso, D. L. *et al.* Two Pairs of ON and OFF Retinal Ganglion Cells Are Defined by Intersectional Patterns of Transcription Factor Expression. *Cell Rep* **15**, 1930-1944, doi:10.1016/j.celrep.2016.04.069 (2016).

4 Masland, R. H. The neuronal organization of the retina. *Neuron* **76**, 266-280, doi:10.1016/j.neuron.2012.10.002 (2012).

5 Sanes, J. R. & Masland, R. H. The types of retinal ganglion cells: current status and implications for neuronal classification. *Annu Rev Neurosci* **38**, 221-246, doi:10.1146/annurev-neuro-071714-034120 (2015).

6 Duan, X. *et al.* Subtype-Specific Regeneration of Retinal Ganglion Cells following Axotomy: Effects of Osteopontin and mTOR Signaling. *Neuron* **85**, 1244-1256, doi:10.1016/j.neuron.2015.02.017 (2015).

7 Brecha, N. C., Oyster, C. W. & Takahashi, E. S. Identification and characterization of tyrosine hydroxylase immunoreactive amacrine cells. *Invest Ophthalmol Vis Sci* **25**, 66-70 (1984).

8 Sümbül, U. *et al.* A genetic and computational approach to structurally classify neuronal types. *Nat Commun* **5**, 3512, doi:10.1038/ncomms4512 (2014).

9 Badea, T. C. & Nathans, J. Morphologies of mouse retinal ganglion cells expressing transcription factors Brn3a, Brn3b, and Brn3c: analysis of wild type and mutant cells using genetically-directed sparse labeling. *Vision Res* **51**, 269-279, doi:10.1016/j.visres.2010.08.039 (2011).

10 Wang, S. W. *et al.* Brn3b/Brn3c double knockout mice reveal an unsuspected role for Brn3c in retinal ganglion cell axon outgrowth. *Development* **129**, 467-477 (2002).

11 Nadal-Nicolás, F. M. *et al.* Whole number, distribution and co-expression of brn3 transcription factors in retinal ganglion cells of adult albino and pigmented rats. *PLoS One* **7**, e49830, doi:10.1371/journal.pone.0049830 (2012).

12 Pak, W., Hindges, R., Lim, Y. S., Pfaff, S. L. & O'Leary, D. D. Magnitude of binocular vision controlled by islet-2 repression of a genetic program that specifies laterality of retinal axon pathfinding. *Cell* **119**, 567-578, doi:10.1016/j.cell.2004.10.026 (2004).

13 Triplett, J. W. *et al.* Dendritic and axonal targeting patterns of a genetically-specified class of retinal ganglion cells that participate in image-forming circuits. *Neural Dev* **9**, 2, doi:10.1186/1749-8104-9-2 (2014).

14 Kim, I. J., Zhang, Y., Meister, M. & Sanes, J. R. Laminar restriction of retinal ganglion cell dendrites and axons: subtype-specific developmental patterns revealed with transgenic markers. *J Neurosci* **30**, 1452-1462, doi:10.1523/JNEUROSCI.4779-09.2010 (2010).

15 Estevez, M. E. *et al.* Form and function of the M4 cell, an intrinsically photosensitive retinal ganglion cell type contributing to geniculocortical vision. *J Neurosci* **32**, 13608-13620, doi:10.1523/JNEUROSCI.1422-12.2012 (2012).

16 Zhang, Y., Kim, I. J., Sanes, J. R. & Meister, M. The most numerous ganglion cell type of the mouse retina is a selective feature detector. *Proc Natl Acad Sci U S A* **109**, E2391-2398, doi:10.1073/pnas.1211547109 (2012).

17 El-Danaf, R. N. & Huberman, A. D. Characteristic patterns of dendritic remodeling in early-stage glaucoma: evidence from genetically identified retinal ganglion cell types. *J Neurosci* **35**, 2329-2343, doi:10.1523/JNEUROSCI.1419-14.2015 (2015).

18 Shi, M. *et al.* Genetic interactions between Brn3 transcription factors in retinal ganglion cell type specification. *PLoS One* **8**, e76347, doi:10.1371/journal.pone.0076347 (2013).

19 Mao, C. A. *et al.* T-box transcription regulator Tbr2 is essential for the formation and maintenance of Opn4/melanopsin-expressing intrinsically photosensitive retinal ganglion cells. *J Neurosci* **34**, 13083-13095, doi:10.1523/JNEUROSCI.1027-14.2014 (2014).

20 Kay, J. N. *et al.* Retinal ganglion cells with distinct directional preferences differ in molecular identity, structure, and central projections. *J Neurosci* **31**, 7753-7762, doi:10.1523/JNEUROSCI.0907-11.2011 (2011).

21 Baden, T. *et al.* The functional diversity of retinal ganglion cells in the mouse. *Nature* **529**, 345-350, doi:10.1038/nature16468 (2016).

22 Sajgo, S. *et al.* Molecular codes for cell type specification in Brn3 retinal ganglion cells. *Proc Natl Acad Sci U S A* **114**, E3974-E3983, doi:10.1073/pnas.1618551114 (2017).

23 Rodriguez, A. R., de Sevilla Müller, L. P. & Brecha, N. C. The RNA binding protein RBPMS is a selective marker of ganglion cells in the mammalian retina. *J Comp Neurol* **522**, 1411-1443, doi:10.1002/cne.23521 (2014).

24 Piri, N., Kwong, J. M., Song, M. & Caprioli, J. Expression of hermes gene is restricted to the ganglion cells in the retina. *Neurosci Lett* **405**, 40-45, doi:10.1016/j.neulet.2006.06.049 (2006).

25 Kwong, J. M., Caprioli, J. & Piri, N. RNA binding protein with multiple splicing: a new marker for retinal ganglion cells. *Invest Ophthalmol Vis Sci* **51**, 1052-1058, doi:10.1167/iovs.09-4098 (2010).

26 Quina, L. A. *et al.* Brn3a-expressing retinal ganglion cells project specifically to thalamocortical and collicular visual pathways. *J Neurosci* **25**, 11595-11604, doi:10.1523/JNEUROSCI.2837-05.2005 (2005).

27 Jiang, Y. *et al.* Transcription factors SOX4 and SOX11 function redundantly to regulate the development of mouse retinal ganglion cells. *J Biol Chem* **288**, 18429-18438, doi:10.1074/jbc.M113.478503 (2013).

28 Chang, K. C. *et al.* Novel Regulatory Mechanisms for the SoxC Transcriptional Network Required for Visual Pathway Development. *J Neurosci* **37**, 4967-4981, doi:10.1523/JNEUROSCI.3430-13.2017 (2017).

29 Kuwajima, T., Soares, C. A., Sitko, A. A., Lefebvre, V. & Mason, C. SoxC Transcription Factors Promote Contralateral Retinal Ganglion Cell Differentiation and Axon Guidance in the Mouse Visual System. *Neuron* **93**, 1110-1125.e1115, doi:10.1016/j.neuron.2017.01.029 (2017).

30 Trakhtenberg, E. F. *et al.* Serotonin receptor 2C regulates neurite growth and is necessary for normal retinal processing of visual information. *Dev Neurobiol*, doi:10.1002/dneu.22391 (2016).

31 Martins, J. *et al.* Activation of Neuropeptide Y Receptors Modulates Retinal Ganglion Cell Physiology and Exerts Neuroprotective Actions In Vitro. *ASN Neuro* **7**, doi:10.1177/1759091415598292 (2015).

32 Kim, I. J., Zhang, Y., Yamagata, M., Meister, M. & Sanes, J. R. Molecular identification of a retinal cell type that responds to upward motion. *Nature* **452**, 478-482, doi:10.1038/nature06739 (2008).

33 Krishnaswamy, A., Yamagata, M., Duan, X., Hong, Y. K. & Sanes, J. R. Sidekick 2 directs formation of a retinal circuit that detects differential motion. *Nature* **524**, 466-470, doi:10.1038/nature14682 (2015).

34 Cherry, T. J. *et al.* NeuroD factors regulate cell fate and neurite stratification in the developing retina. *J Neurosci* **31**, 7365-7379, doi:10.1523/JNEUROSCI.2555-10.2011 (2011).

35 Santone, R. *et al.* Gene expression and protein localization of calmodulin-dependent phosphodiesterase in adult rat retina. *J Neurosci Res* **84**, 1020-1026, doi:10.1002/jnr.21009 (2006).

36 Ivanova, E., Lee, P. & Pan, Z. H. Characterization of multiple bistratified retinal ganglion cells in a purkinje cell protein 2-Cre transgenic mouse line. *J Comp Neurol* **521**, 2165-2180, doi:10.1002/cne.23279 (2013).

37 de Melo, J. *et al.* Dlx1 and Dlx2 function is necessary for terminal differentiation and survival of late-born retinal ganglion cells in the developing mouse retina. *Development* **132**, 311-322, doi:10.1242/dev.01560 (2005).

38 Ding, Q. *et al.* BARHL2 differentially regulates the development of retinal amacrine and ganglion neurons. *J Neurosci* **29**, 3992-4003, doi:10.1523/JNEUROSCI.5237-08.2009 (2009).

39 Mao, C. A. *et al.* Eomesodermin, a target gene of Pou4f2, is required for retinal ganglion cell and optic nerve development in the mouse. *Development* **135**, 271-280, doi:10.1242/dev.009688 (2008).

40 Jin, K., Jiang, H., Mo, Z. & Xiang, M. Early B-cell factors are required for specifying multiple retinal cell types and subtypes from postmitotic precursors. *J Neurosci* **30**, 11902-11916, doi:10.1523/JNEUROSCI.2187-10.2010 (2010).

41 Sweeney, N. T., Tierney, H. & Feldheim, D. A. Tbr2 is required to generate a neural circuit mediating the pupillary light reflex. *J Neurosci* **34**, 5447-5453, doi:10.1523/JNEUROSCI.0035-14.2014 (2014).

42 Chew, K. S., Schmidt, T. M., Rupp, A. C., Kofuji, P. & Trimarchi, J. M. Loss of gq/11 genes does not abolish melanopsin phototransduction. *PLoS One* **9**, e98356, doi:10.1371/journal.pone.0098356 (2014).

43 Rivlin-Etzion, M. *et al.* Transgenic mice reveal unexpected diversity of on-off direction-selective retinal ganglion cell subtypes and brain structures involved in motion processing. *J Neurosci* **31**, 8760-8769, doi:10.1523/JNEUROSCI.0564-11.2011 (2011).

44 Peng, Y. R. *et al.* Satb1 Regulates Contactin 5 to Pattern Dendrites of a Mammalian Retinal Ganglion Cell. *Neuron* **95**, 869-883.e866, doi:10.1016/j.neuron.2017.07.019 (2017).

45 Groman-Lupa, S., Adewumi, J., Park, K. U. & Brzezinski Iv, J. A. The Transcription Factor Prdm16 Marks a Single Retinal Ganglion Cell Subtype in the Mouse Retina. *Invest Ophthalmol Vis Sci* **58**, 5421-5433, doi:10.1167/iovs.17-22442 (2017).

46 Watanabe, M., Rutishauser, U. & Silver, J. Formation of the retinal ganglion cell and optic fiber layers. *J Neurobiol* **22**, 85-96, doi:10.1002/neu.480220109 (1991).

47 Trakhtenberg, E. F. *et al.* Cell types differ in global coordination of splicing and proportion of highly expressed genes. *Sci Rep* **6**, 32249, doi:10.1038/srep32249 (2016).

48 Macosko, E. Z. *et al.* Highly Parallel Genome-wide Expression Profiling of Individual Cells Using Nanoliter Droplets. *Cell* **161**, 1202-1214, doi:10.1016/j.cell.2015.05.002 (2015).

49 Vaquerizas, J. M., Kummerfeld, S. K., Teichmann, S. A. & Luscombe, N. M. A census of human transcription factors: function, expression and evolution. *Nat Rev Genet* **10**, 252-263, doi:10.1038/nrg2538 (2009).

50 Dacey, D. M. & Packer, O. S. Colour coding in the primate retina: diverse cell types and cone-specific circuitry. *Curr Opin Neurobiol* **13**, 421-427 (2003).

51 Chang, L., Breuninger, T. & Euler, T. Chromatic coding from cone-type unselective circuits in the mouse retina. *Neuron* **77**, 559-571, doi:10.1016/j.neuron.2012.12.012 (2013).

52 Lovén, J. *et al.* Revisiting global gene expression analysis. *Cell* **151**, 476-482, doi:10.1016/j.cell.2012.10.012 (2012).

53 Li, S. *et al.* Promoting axon regeneration in the adult CNS by modulation of the melanopsin/GPCR signaling. *Proc Natl Acad Sci U S A* **113**, 1937-1942, doi:10.1073/pnas.1523645113 (2016).

54 Belin, S. *et al.* Injury-induced decline of intrinsic regenerative ability revealed by quantitative proteomics. *Neuron* **86**, 1000-1014, doi:10.1016/j.neuron.2015.03.060 (2015).

55 Masland, R. H. & Tauchi, M. The cholinergic amacrine cell. *Trends in Neurosciences* **9**, 218-223, doi:10.1016/0166-2236(86)90062-7 (1986).

56 Iwamoto, K., Mata, D., Linn, D. M. & Linn, C. L. Neuroprotection of rat retinal ganglion cells mediated through alpha7 nicotinic acetylcholine receptors. *Neuroscience* **237**, 184-198, doi:10.1016/j.neuroscience.2013.02.003 (2013).

57 Bansal, A. *et al.* Mice lacking specific nicotinic acetylcholine receptor subunits exhibit dramatically altered spontaneous activity patterns and reveal a limited role for retinal waves in forming ON and OFF circuits in the inner retina. *J Neurosci* **20**, 7672-7681 (2000).
