# Supplementary material for: Single cell transcriptome profiling of retinal ganglion cells identifies cellular subtypes
Source: Nat Commun. 2018 Jul 17;9:2759. doi: 10.1038/s41467-018-05134-3 (PMC6050223; doi:10.1038/s41467-018-05134-3)
Supplement: Supplementary file 2 — Description of Additional Supplementary Files [file 41467_2018_5134_MOESM2_ESM.pdf]

Description of Additional Supplementary Files

File Name: Supplementary Data 1
Description: A data matrix containing uniquely enriched genes and their respective normalized expression (*NE*) values per cluster. *NE* values are highlighted in beige in the columns that correspond to the clusters in which they are enriched. Enriched genes criteria: expression > 1.8-fold relative to every other cluster, and minimal average expression > 0.05 *NE*. Up to 5 top genes per cluster with *p*-value < 0.05 are specified in Fig. 4. The list here, however, also includes genes enriched only by the first two criteria -- even if *p*-value > 0.05.

File Name: Supplementary Data 2
Description: A list of RGC housekeeping and subtype-enriched transcription factors and their respective expression levels.
